# Explainable AI for Cotton Leaf Disease Classification: A Metaheuristic‐Optimized Deep Learning Approach

**DOI:** 10.1002/fsn3.70658

**Published:** 2025-07-22

**Authors:** Gurjot Kaur, Fuad Ali Mohammed Al‐Yarimi, Salil Bharany, Ateeq Ur Rehman, Seada Hussen

**Affiliations:** ^1^ Chitkara University Institute of Engineering and Technology Chitkara University Rajpura Punjab India; ^2^ Applied College of Mahail Aseer King Khalid University Saudi Arabia; ^3^ School of Computing Gachon University Republic of Korea; ^4^ Department of Electrical Power Adama Science and Technology University Adama Ethiopia

**Keywords:** cotton leaf disease classification, deep learning, explainable artificial intelligence, genetic algorithm, smart agriculture, SMOTE

## Abstract

Cotton leaf diseases significantly impact global cotton yield and quality, threatening the livelihoods of millions of farmers. Traditional diagnostic methods are often slow, subjective, and unsuitable for large‐scale agricultural monitoring. This study proposes an interpretable and efficient deep learning (DL) framework for the accurate classification of cotton leaf diseases using a hybrid architecture that combines EfficientNetB3 and InceptionResNetV2. The system demonstrates excellent performance, achieving 98.0% accuracy, 98.1% precision, 97.9% recall, an F1‐score of 98.0%, and an AUC‐ROC of 0.9992. Minimal overfitting was observed, with low training and validation losses and high per‐class performance, even in visually similar disease cases such as bacterial blight and target spot. In addition to strong predictive accuracy, the framework incorporates explainable AI (XAI) techniques, including LIME and SHAP, to enhance model transparency. These tools highlight the key visual features used in predictions, providing valuable insights for agronomists and improving trust in AI‐based systems. The model is lightweight and scalable, making it deployable on mobile or edge devices for real‐time field applications. Overall, this research demonstrates the potential of combining transfer learning and XAI to develop reliable, interpretable, and field‐ready diagnostic tools for precision agriculture.

## Graphical abstract

A hybrid deep learning (DL) model combining EfficientNetB3 and InceptionResNetV2 achieved 98% accuracy in classifying six types of cotton leaf diseases. Genetic Algorithm (GA) optimization improved model generalization, while LIME and SHAP provided interpretable visual and feature‐based explanations, enhancing trust among users. The model is lightweight, deployable on mobile devices, and shows high performance even on visually similar disease categories, supporting real‐time precision agriculture.

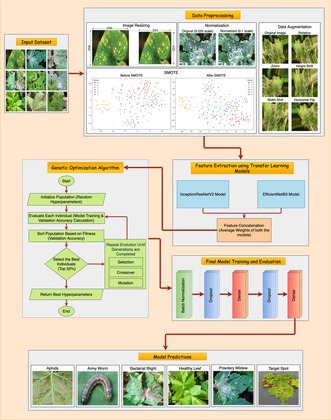

## 1 Introduction

Cotton stands as a leading cash crop internationally and serves primarily as a raw material input for textile manufacturing (Tripathy 2021). These top cotton‐producing nations, India, China, the United States, and Pakistan, help the economic development of emerging and developed countries worldwide. The worldwide cotton trade system needs millions of farmers to work, supporting significant international business operations. Leaf diseases are more destructive than other crop stress factors affecting cotton production. Compared with other diseases, bacterial blight, Alternaria leaf spot, and leaf curl virus diseases reduce both cotton plant quality and harvest quantity. These illnesses require early detection and treatment because they cause significant financial damage to farmers (Patki and Sable 2016). Farmer and agronomist inspections of fields remain a time‐consuming and imprecise leaf disease detection method for regular use (Sallom and Alabboud 2023). An automated disease classification system helps farmers take necessary actions faster, which requires system development. Deep learning (DL) technology and artificial intelligence tools accurately detect plant diseases from leaf images at a very high level of precision. DL hybrid models and convolutional neural networks (CNNs) achieve good results when detecting plant diseases. These models face a significant disadvantage because users cannot see how they make decisions, which leads farmers and agricultural experts to distrust their output. Explainable AI technology has become vital for agrarian systems because of its limitations. The XAI methods of SHAP, Grad‐CAM, and LIME allow AI models to become transparent by showing where and how they make decisions. This research integrates XAI technology into DL‐based cotton leaf disease classification to improve model transparency and make better decisions in farming operations (Gulhane and Gurjar 2011).

Several visible plant symptoms appear during leaf diseases that negatively affect plant health and reduce crop production. Cotton leaf diseases mainly include bacterial blight, Alternaria leaf spot, leaf curl virus, and fungi. Different diseases display clear signs that healthcare professionals use to identify and treat problems early. The infected areas of plants first look water‐damaged before turning completely dead, decreasing the strength and leaf coverage of the plants. Alternaria leaf spots form distinct dark brown circular lesions with rings on the leaves, while yellow halos develop around them, which reduces leaf photosynthesis. Cotton leaf curl virus (CLCuV) causes the leaves of cotton plants to curl and twist while thickening the veins as growth slows and the crop yield decreases. Certain fungal diseases, such as Verticillium and Fusarium wilt, affect cotton plants through their vascular system and cause the death of cells, which results in yellowing and wilting of plant parts. Discovering symptoms at the beginning allows proper control steps to start on time. Traditional identification methods involving visual checking take too long for workers to perform, and they make errors. DL models detect disease symptoms automatically, which helps identify and classify plant damage patterns effectively (Briddon 2003). Multiple types of infection and environmental pressures across bacteria, fungi, viruses, and extreme weather conditions trigger leaf diseases in cotton plants. Identifying disease origins helps us develop successful ways to prevent and control these health problems. The microscopic pathogen *Xanthomonas citri pv. Malvacearum* develops well in warm and humid surroundings. Continuous rainwater and infected cotton plant waste are distributed across rice field boundaries in tropical and subtropical cotton‐growing zones. The fungal organisms *Alternaria macrospora* and *Alternaria alternata* infect weak plants primarily when humidity and temperature variations are high. CLCuV transmits the disease when whiteflies carry it from one plant to another. Viral infection damages cotton production by slowing plant development and warping the leaves. Defects in the ground, poor nutrition, heavy pesticide use, and unpredictable weather weaken plants, increasing their susceptibility to infection. Improved farming methods, specific crop types, and quick actions can help prevent these factors from damaging plants. AI‐based disease detection systems improve our ability to control and stop plant problems when linked with agricultural farming techniques (Sattar et al. 2013).

Effective cotton leaf disease control needs actions from farming methods, biological controls, pesticide treatments, and innovative technology solutions. The growth of cotton plants that resist diseases protects them from regular infections. Winter planting of plants that do not support disease helps both disrupt pathogen reproduction and reduce harmful plant residue. Good sanitation practices require that infected plant materials be removed and destroyed to prevent the spread of fungal and bacterial spores. When used as biological controls, *Trichoderma* and *Bacillus* microorganisms fight disease‐causing fungi and increase plant immunity. Whitefly protection forms an essential part of IPM approaches to contain the spread of CLCuV. Scientists use reliable disease forecasting models to help farmers prevent outbreaks through proper pesticide treatment at the right time. New technological systems, such as AI and remote sensing, allow farmers to detect diseases early and treat affected areas better, which saves crops. The incorporation of prevention methods with eco‐friendly farming systems allows cotton farmers to grow more and lower their risk while maintaining the health of the environment (Farooq et al. 2011). DL tools for plant disease detection have become more effective, but many lack explanations that people in agriculture need to rely on them. Despite their high accuracy standard, DL networks such as CNNs work as mystery systems and show users how decisions are made. When AI systems for disease detection cannot show their reasons for predictions, farmers and experts cannot trust or use them in real farming situations. DL platforms require extensive work because they must operate precisely and quickly. Advanced detection technology requires significant computer resources that hinder its use in fast agricultural work and resource‐limited areas. Model selection and data enhancement help improve quality, yet substantial testing is needed. The model accuracy decreases when uneven training material and changing environmental situations are encountered. Research demands a DL system that can perform reliably and transparently while producing high‐quality results (Ghaiwat and Arora 2016). With our study, we produce a comprehensible DL model to identify cotton leaf disease that surpasses the limited capability of simple AI devices. XAI methods that allow simple explanations of AI to agronomists and farmers are included in the project. Optimization methods developed in this research provide more accurate disease detection at faster rates because the hyperparameters optimal settings are found. The optimization approaches will reduce model stability and the processing requirements. The presence of XAI technology will demonstrate how users can achieve results through their systems, creating a sense of trust as well as straightforwardness of use. Our technique also has to be tested adequately against the industry so that it demonstrates the level of its accuracy alongside how effective and efficient it is. The idea of this work was to create functional AI disease management tools and reduce the gap between DL systems and agricultural needs (Ramya and Kalimuthu 2024).

Despite the technical focus of this research on DL and explainable AI, its ultimate goal is to contribute to sustainable agriculture and food security, which are critical pillars of global nutrition systems. Cotton is not only essential for textile industries but also plays a key role in rural economies and livelihoods. Early disease detection through AI‐powered diagnostic systems can substantially reduce crop losses, ensure higher yield quality, and lower dependency on chemical pesticides, thereby promoting safer and more sustainable agricultural practices. These outcomes align closely with the broader objectives of *Food Science & Nutrition*, particularly in relation to enhancing food supply chains, reducing agroeconomic vulnerability, and promoting technological innovation in crop health monitoring. By enabling precision intervention at the farm level, this research supports the nutritional and economic stability of millions of cotton‐farming communities globally.

The primary contributions of this research are as follows:

1. This research develops a novel DL model for cotton leaf disease identification, plus XAI features to make system operations more straightforward to understand.
2. The research tests model hyperparameters via metaheuristic optimization methods, which yield greater accuracy and optimization for real‐world agricultural tasks without DL techniques.
3. The researchers built SHAP and LIME methods into the model to make decisions more straightforward to understand, which improved trust and usability among farmers and agronomists.
4. The study compares its proposed approach to other AI models to show how well it detects patterns while remaining easy to understand at an efficient speed.

Research Questions: This study was guided by the following research questions:

- RQ1: Can a hybrid EfficientNetB3 + InceptionResNetV2 architecture optimized using a Genetic Algorithm (GA) outperform conventional and state‐of‐the‐art models in multiclass cotton leaf disease detection?
- RQ2: Can Explainable AI (XAI) tools such as SHAP and LIME offer meaningful interpretations of the model predictions, thereby increasing trust and transparency in practical agricultural applications?

The next sections of this research study describe every part of the text. In Section II, the research introduces the primary features of cotton leaf diseases, their origins and presentations, and their influence on the global cotton market. The text defines the requirements of disease detection technologies and demonstrates how AI enhances farming accuracy. Section III analyzes existing articles about DL systems that identify cotton diseases. Section IV explains our research methods, which include designing a DL network architecture, followed by describing the dataset and preprocessing steps before testing different metaheuristic algorithms to enhance the model's performance. Section V shows the experimental outcomes by analyzing how the proposed solution outperforms standard models in all performance aspects and displays the benefits offered by XAI tools. The research finds significant results concerning agricultural applications while presenting deployment challenges in Section VI, yet it underlines why explainable AI should help produce working outcomes. The report concludes in Section VII with a summary of the results and suggestions for the next steps for making AI better at farm‐level disease identification.

## 2 Literature Review

Accurate detection of cotton leaf diseases is essential for preserving crop yield, ensuring quality, and promoting sustainable agricultural practices. Conventional disease diagnosis, which relies on manual inspection by experts, is often time‐intensive, prone to human error, and unsuitable for large‐scale farming. Recent advancements in DL, particularly in transfer learning, CNNs, transformer‐based architectures, and XAI, have significantly improved the automation, scalability, and transparency of disease classification systems. The modern AI‐based strategies minimize the need to rely on expert knowledge and can provide quicker, more uniform, and interpretable disease detection capacities. This part amounts to a thorough survey of up‐to‐date cotton leaf disease detection systems with an examination of their technical approaches and performance, advantages, drawbacks, and the possible emergence of optimization and interpretability.

To demonstrate the role of AI in agriculture to identify plant issues, Islam et al. (2023) identified cotton leaf disease using a DL system. As shown in their results, less than 70 to 80% of the cotton diseases are leaf‐based, so there is a need to adopt automatic detection machinery at an early stage. They applied bad models such as VGG‐16, VGG‐19, Inception‐V3, and Xception to perform transfer learning that helped identify cotton leaf diseases. The experimental results show that the Xception model reaches 98.70% accuracy for actual use applications. The system became part of a smart web application that allows users to spot diseases right away to help improve cotton farming (Islam et al. 2023). By spotting cotton leaf diseases earlier helped farmers grow more crops while reducing the use of pesticide chemicals. Their work combined artificial intelligence methods to process cotton leaf pictures and develop automatic disease rating systems. The examination system identified infected leaves early to decrease yield loss before damage became extensive. The accuracy, precision, F1 score, and recall scores of these models were tested. The proposed approach achieved 97% accuracy in detecting cotton crop diseases ahead of time, according to experimental tests (Salot et al. 2024).

Bishshash et al. (2024) established complete information on cotton leaf disease that helps farmers and scientists improve farm techniques and protect crops. Their team gathered 2137 genuine field images plus 7000 image additions from October 2023 to January 2024 to create eight disease and agricultural strain categories. This dataset helps DL models perform better by detecting diseases autonomously. The Inception V3 neural network design achieved 96.03% correct predictions, which demonstrates that this dataset can be used to develop accurate AI‐controlled disease‐tracking systems for cotton agriculture (Bishshash et al. 2024). Nagpal and Goel (2024) investigated how machine learning could automatically identify plant diseases in maize and cotton leaves through technology improvement. They analyzed their study through three hyperparameter tuning methods using 2543 maize and 1443 cotton leaf images. The scientists developed dynamic accelerated memory‐based PSO (DAMPSO) as a new method to optimize system parameters. The tests confirmed that DAMPSO outperforms the other methods with 95% accuracy, and XGBoost was used to classify plant diseases (Nagpal and Goel 2024).

Iqbal et al. (2024) revealed that DL plays a critical role in smart agriculture, specifically plant disease diagnosis automation, through their research. Their research team applied EfficientNet, which specializes in single‐label image patch classification, and trained the model on a collection of 20,000 cotton leaf pictures. The model produced results that were verified through typical quality measures and offered 88% recall along with 89% precision and 89% F1‐scoring. This research proves that deep transfer learning enables the trained models to be more applicable in terms of the identification of agricultural diseases and can provide new technology in farming (Iqbal et al. 2024). Meena et al. (2024) were able to create such a system, which can automatically detect the presence of cotton diseases utilizing a CNN and the use of GLCM to extract features. The GLCM identifies the texture characteristics of cotton plant images that help detect different types of diseases. By processing images, the CNN system determines their category as either CLCV infection, bacterial blight defoliation, or normal plant appearance. The combined GLCM CNN method successfully identified cotton diseases early through accurate detection, with an 87% success rate and precision reaching as high as 0.88 (Meena et al. 2024).

Akbar et al. (2024) studied how to solve cotton lesion detection automation issues such as unequal class distribution, small dataset availability, and evaluation metric requirements. Their research suggested the use of GAN‐based DL to add virtual data samples to fix problems caused by unequal data distributions. Their method uses VGG16, Inception V3, and ResNet50 architectures to generate better results and handle increasing amounts of data. The suggested method yielded top results from the evaluation of a public dataset that contained seven disease categories and one control group. It achieved a 95% accurate diagnosis and 98% F1 score, which surpassed those of the present state‐of‐the‐art benchmarks (Akbar et al. 2024). Ahmad, Qadri, and Akhtar (2024) and Ahmad, Qadri, Akhtar, and Nawaz (2024) stated that Ahmad and colleagues developed a framework that uses multiple cameras to identify the impact of the cotton leaf curl virus at several levels of severity. They analyzed three unique datasets, including regular digital images alongside multispectral images and their combination. Harvesting the best results required merging and processing 30 texture features from digital images and five spectral features from MSR5 data. We selected the random forest and multilayer perceptron machine learning models among the four classifiers for this study. Combining both digital and multispectral data produced 96.31% accurate results, which exceeded the single dataset results of 81.26% and 91.17%, respectively. This confirmed that the fusion method effectively depicts very early CLCuV infection (Ahmad, Qadri, and Akhtar 2024).

Ahmad, Qadri, and Akhtar (2024) and Ahmad, Qadri, Akhtar, and Nawaz (2024) created an automatic system that detects CLCuV through machine learning techniques. They obtained healthy, mild, and severe infection leaf examples from cotton fields and enhanced all the images via a common processor. The research team employed histogram GLCM and RLM to extract valuable features from their samples. The process of selecting important features uses Fisher, probability of error, average correlation, and mutual information techniques to lower computer processing demands. RF proved to be the best classifier for CLCuV detection, with an 87.54% success rate (Ahmad, Qadri, Akhtar, and Nawaz 2024). Nimbhore et al. (2024) created an MDFC‐based model to classify cotton crop diseases through image analysis steps. Because of bilateral filtering and MDFC segmentation, the model works best at identifying plant disorders. PHOG, LDTP, and statistical feature sets undergo extraction before Bi‐GRU and modified RNN classification. The suggested method showed better results than the other models because it reached 0.968 specificity, outperforming the CNN at 0.865 and the LSTM at 0.876. Additionally, VGG16 scored 0.861. For the detection of cotton diseases, the combination of DL techniques achieved the highest accuracy (Nimbhore et al. 2024).

To address the problem of poor performance in different farming environments, Shao et al. (2024) have come up with CANnet, a DL model for the detection of agricultural diseases. In their system, they introduced two other elements, namely the reception field space channel (RFSC) to amplify spatial attention and precise coordinate attention (PCA) to avoid overduplication of features. To develop superior solutions, standard MLP networks were substituted with network classifiers using *Kolmogorov‐Arnold* networks. Their approach was proven to be the best among the others, as the experimental works yielded positive results regarding correct test results. It has obtained 96.3% correct identifications on a verifier who created the study set and 98.6% correct identifications on a set of databases, which researchers make available to one another publicly (Shao et al. 2024). The modified DL model that was developed by Shahid et al. (2024) helps to identify diseases in cotton plantations using AI and image processing technologies. Continuous wavelet transform and fast Fourier transform were used to make GoogLeNet give the best individual model performance at 93.4% accuracy in this study. The framework using the CWT features attained an accuracy of 98.4% through its ensemble learning method. Our investigation was successful, as it revealed that feature extraction via the scalogram technique coupled with DL is effective when identifying cotton diseases and augmenting production outputs (Shahid et al. 2024).

The Swin transformer platform improved by Zhang, Zhu, et al. (2024) and Zhang, Lin, et al. (2024) took the approach of classifying the type of cotton pests even in the absence of a clear distinction between visual backgrounds. With residual modules and skip connections, the model performs better at detecting pest characteristics to reduce the number of classification errors. The training was done through a set of 2705 images of cotton pests, representing cotton aphid, cotton mirid, as well as cotton leaf mites. The new model scored 97.4%, which exceeded that of the old model and performed better than VGG11, ResNet18, and MobileNetV2. This model is capable of inference on edge devices and UAVs due to its efficiency and precision, and so it can be used to detect pests in the agricultural field in real time (Zhang, Zhu, et al. 2024). Li et al. (2024) created a powerful CFNet‐VoV‐GCSP‐LSKNet‐YOLOv8s model to identify cotton diseases and insects in their natural habitat and address performance limitations of simple models of detection. The study integrates both CFNet scale‐level feature fusion and VoV‐GCSP in a bid to maintain the trade‐off between precision and inference speed, as well as the LSKNet network of seeking small displacements and the efficient convergence support of the XIoU loss. The model showed a mean average precision of 93.7% that surpassed the YOLO v5s, YOLOX, and the Faster R‐CNN models in the range of 21.8%–1.2%. The precision and recall were 89.9 and 90.7, respectively. In this study, precision farming has been advanced as it stores and identifies automatically the pest and disease issues in cotton fields (Li et al. 2024). He et al. (2024) proposed YOLOv9‐LSBN to detect crops in a field in terms of bugs and diseases. The model also adopts the strategy of RepLanLsk to extract more suitable features and runs BIFPN weight modification as a method of producing the most suitable feature combinations. The experiment has performed better than the other YOLOv models, resulting in 93% accuracy, 92.4% recall, and 96.4% average precision. The study proves that the model is effective in the presence of background and can detect pests accurately and minimize mistakes, things to the advantage of agriculture pest monitoring systems (He et al. 2024). These studies have been summarized in Table 1.

**TABLE 1:** Literature review.

Reference No.	Technique used	Advantages	Disadvantages	Results
Islam et al. (2023)	Transfer Learning (VGG‐16, VGG‐19, Inception‐V3, Xception)	High performance in real‐time detection	Limited to TL models, may need further optimization	Xception achieved the highest accuracy (98.70%)
Salot et al. (2024)	Machine Learning and Deep Learning Techniques	Early disease detection and efficient classification	It relies on deep learning models, which are computationally expensive	Achieved 97% accuracy in early disease detection
Bishshash et al. (2024)	Inception V3 model	A comprehensive dataset supports deep learning applications	Dataset availability is limited to specific cotton diseases	96.03% accuracy
Nagpal and Goel (2024)	Machine Learning with Dynamic Accelerated Memory‐Based PSO (DAMPSO)	Optimized hyperparameter tuning for improved classification	High computational cost of DAMPSO tuning	DAMPSO‐based XGBoost achieved the highest accuracy (95%)
Iqbal et al. (2024)	EfficientNet Deep Learning Model	Effective for single‐label classification in smart agriculture	Limited to single‐label classification	EfficientNet achieved a recall of 0.88 and a precision of 0.89.
Meena et al. (2024)	GLCM Texture Features and CNN	Effective texture feature extraction for classification	Limited improvement over other CNN‐based models	The CNN‐GLCM model achieved 87% accuracy
Akbar et al. (2024)	Generative Adversarial Networks (GANs) and Ensemble Learning	Class imbalance reduction using GANs and ensemble learning	GANs require large datasets and a risk of overfitting	Achieved 95% accuracy, F1‐score of 98%
Ahmad, Qadri, and Akhtar (2024)	Machine Vision‐Based Multimodal Fusion Framework	Fused dataset improves detection capabilities	Requires specialized equipment for multispectral imaging	Fused dataset accuracy (96.31%), outperforming individual datasets
Ahmad, Qadri, Akhtar, and Nawaz (2024)	Random Forest	Automated detection using robust feature extraction	GLCM feature extraction can be computationally intensive	Achieved 87.54% accuracy
Nimbhore et al. (2024)	Modified Deep Fuzzy Clustering (MDFC) and Bi‐GRU	The hybrid approach enhances disease classification	Bi‐GRU and RNN models require high processing power	Specificity of 0.968
Shao et al. (2024)	CANnet Deep Learning Model with RFSC & PCA Modules	Superior feature extraction with novel architecture	High model complexity requires significant training data	Achieved 96.3% accuracy on the self‐built dataset, 98.6% on a public dataset
Shahid et al. (2024)	Ensemble Deep Learning with CWT & FFT	Enhanced classification through ensemble learning	FFT features are less effective than CWT in classification	The ensemble learning framework achieved 98.4% accuracy
Zhang, Zhu, et al. (2024)	Improved Swin Transformer with Residual Modules	Enhanced pest classification using a transformer‐based model	Transformer‐based models require significant computational resources	Improved Swin Transformer achieved 97.4% accuracy
Li et al. (2024)	CFNet‐VoV‐GCSP‐LSKNet‐YOLOv8s Model	Improved precision, recall, and feature extraction	Complex model architecture, high computational cost	YOLOv8s variant achieved 93.7% mAP
He et al. (2024)	YOLOv9‐LSBN Model	Better object detection in complex environments	Requires specialized hardware for real‐time deployment	Improved model accuracy to 93%

## 3 Methodology

**FIGURE 1:**
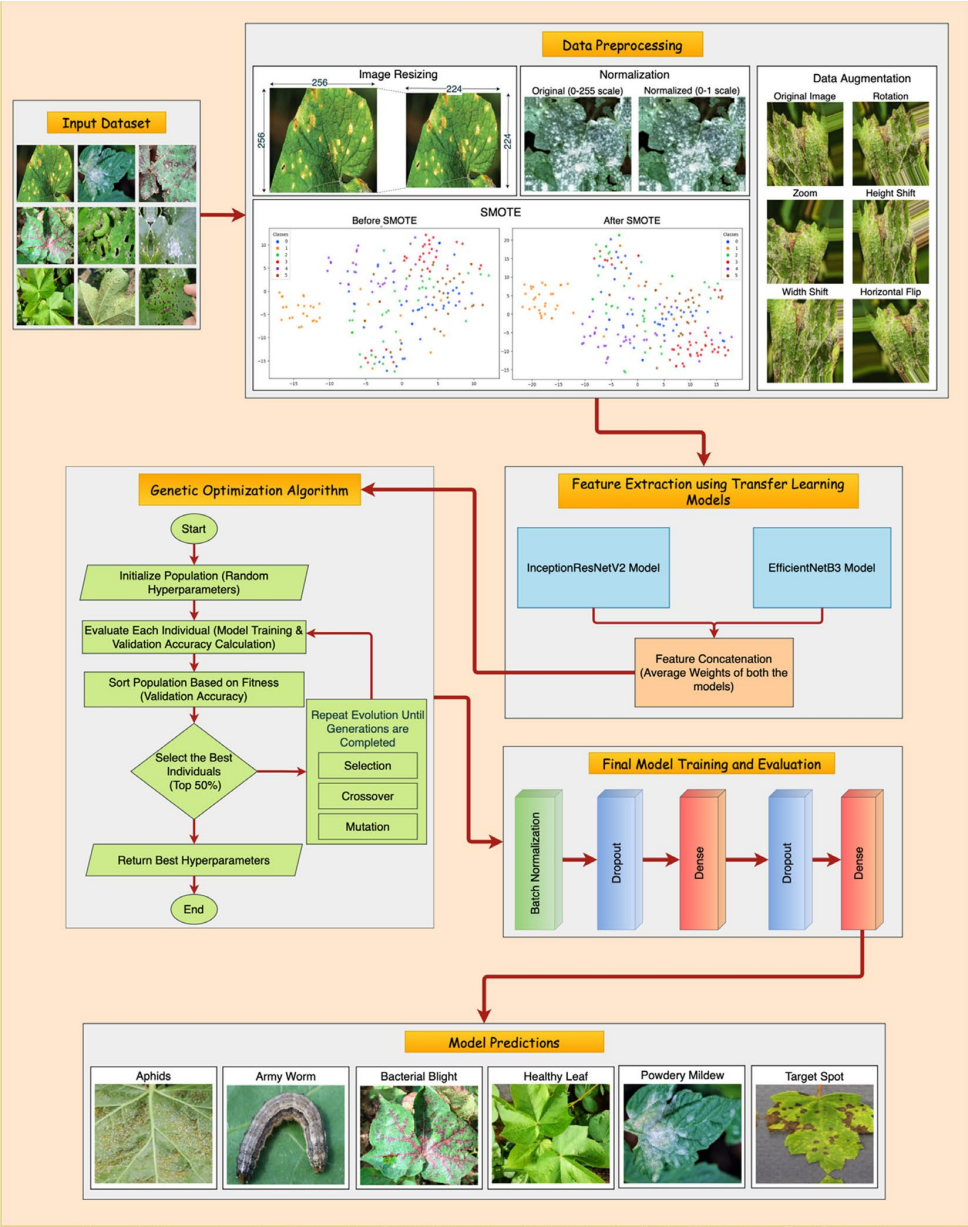
Proposed methodology.

This section shows how to classify cotton leaf diseases through DL and metaheuristic optimization techniques. Our pipeline handles different steps to process data, such as preprocessing it and obtaining relevant features after optimizing hyperparameters and running classification. Before training the model, the dataset receives image filters and then transforms and augments the samples via the synthetic minority oversampling technique (SMOTE) to prepare the model for various scenarios. The system depends on the transfer learning models InceptionResNetV2 and EfficientNetB3 for feature extraction before these extracted features are joined to increase representation accuracy. The genetic optimization algorithm adjusts essential model settings to achieve the best results. The updated information travels through external layers for the final classification of diseases. Figure 1 shows all the steps in the model's working procedure (Bharathi and Manikandan 2025).

### 3.1 Dataset Description

**FIGURE 2:**
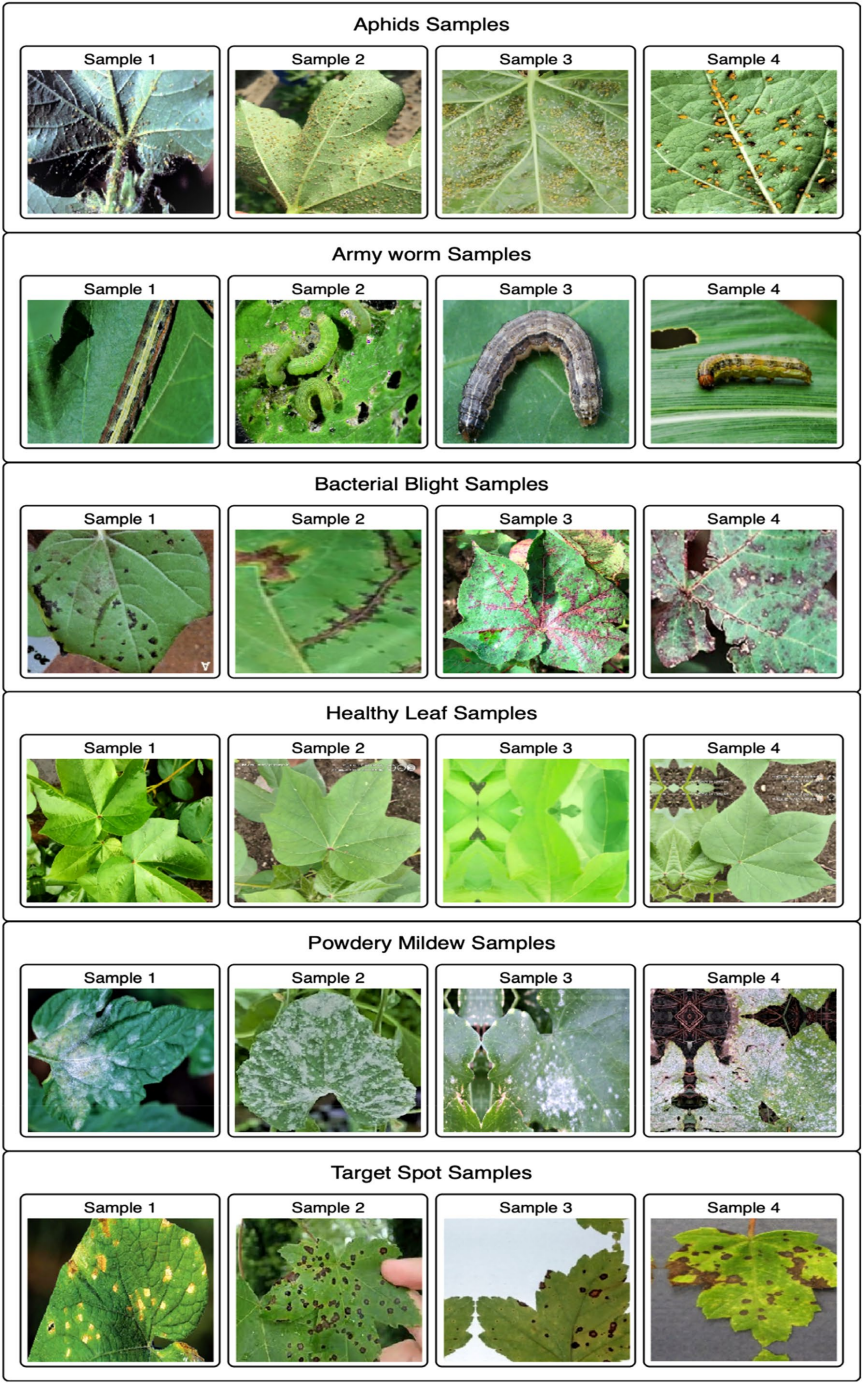
Dataset samples of all classes.

The research utilized cotton leaf images from Kaggle (Kaggle.com 2025), which included 2637 images logically classified into aphid, army worm, bacterial blight, healthy leaf, powdery mildew, and target spot categories. Different light levels and natural environmental factors were utilized to collect 2637 images of contaminated and uninfected cotton leaves, which created suitable training conditions for the model. The images are of high‐definition quality with added labels to enable supervised training. The information is properly organized through distinct folders that represent each disease type. Figure 2 shows the dataset samples of all classes. Aphids that harm cotton yield and plant health inflict damage on the leaves, which presents four affected samples. Small pests known as aphids construct clusters under leaf surfaces where they drain sap fluid and cause foliage discoloration, leaf distortion, and reduced plant growth. The dataset shows high‐quality pictures that demonstrate the diverse effects of bug infestations while revealing different environmental aspects. The divergent elements in the dataset enable training artificial intelligence models to correctly identify and categorize leaves with aphid infestations. Multiple perspective data in the dataset reinforce machine learning applications while permitting exact pest detection, which supports automatic pest management tools for sustainable cotton farming. A total of four cotton leaf samples demonstrated army worm attack effects, which extensively degraded the foliage of cotton plants. The feeding pattern of army worms comprises several destructive traits, including fast spread and heavy consumption, which results in leaf damage patterns that become skeletonized with reduced photosynthetic function. The diverse infestation levels and environmental specifications in the data enable DL algorithms to detect a wide range of army worm damage manifestations. New high‐quality images help machines learn better, which has led to the development of automatic early pest warning systems and targeted agricultural techniques for cotton fields (Ramos et al. 2022).

Bacterial blight ailments in cotton leaves display visible symptoms of water‐soaked lesions and necrotic spots, as well as vein browning. DL models benefit from various stages of bacterial infection visible in the collected samples for advanced training capabilities. The occurrence of this disease is triggered by *Xanthomonas citri pv. malvacearum* spreads through rain splashes and contaminated seeds, which makes early disease detection essential. The detailed images in the dataset enable accurate identification of distinct features, which allows precise classification processes. Using these images, the diagnostic ability of machine learning models can be enhanced to detect cotton diseases immediately and enable effective disease management systems. The healthy leaf samples were used as part of the control data for disease classification model evaluation. The healthy leaf images display clear leaf outlines together with a uniform color scheme with no indications of disease or pest infestation. The images were obtained under different environmental conditions and lighting scenarios, which resulted in better model accuracy and generalizability. Widely used for DL framework training, the healthy leaf dataset helps distinguish between infected and uninfected cotton leaves, thus reducing false alerts during disease detection model operations. Using high‐quality, healthy leaf images during AI‐based agricultural system training produces accurate disease classification capabilities for cotton cultivation (Anwar et al. 2022). Powdery mildew is a fungal disease that results in white powdery mildew growth on cotton leaves, which damages plant photosynthesis and affects the health of the plant system. The infection severity scales from mild spots through multiple stages of infection to complete leaf coverage and appears across the high‐resolution images present in the database. The fungus spreads quickly when humidity is present; therefore, detecting its presence early becomes essential. Because of this diverse sample collection, DL models have become better at detecting fungal infections while discriminating them from other leaf diseases. This leads to improved disease prediction and prompt farmer intervention. The appearance of the target spot in the cotton leaves revealed circular, dark brown spots surrounded by yellow areas. Fungal infection severely harms both cotton production levels and product quality. The dataset contains different infection grades, which helps DL models detect early‐stage infections and separate them from symptoms associated with other pathogens. These detailed images enable researchers to perform feature analysis while dividing images and classifying objects, which leads to superior disease diagnosis and better control methods for sustainable cotton production (Xavier et al. 2019).

### 3.2 Data Preprocessing and Augmentation

The performance, generalizability, and consistency of the proposed model of DL used to classify cotton leaf diseases were improved by a clearly defined preprocessing pipeline that was intended to streamline the process of disease classification. The original dataset was obtained through Kaggle, with high‐resolution pictures being organized into six classes. In order to fix the size of input data and palliate the calculation load, every picture was resized to 224 × 224 pixels. This dimension standardization allows compatibility with the input requirements of EfficientNetB3 and InceptionResNetV2, the two backbone architectures upon which the feature extraction will happen. Min‐max scaling was used as normalization: all values of pixels were transformed to the interval [0, 1]. This step improves upon the convergence of the model and numerical stability when training the model, especially in deep networks where unnormalized data may give gradient instabilities. Several data augmentation methods were used to enhance the generalization of the trained model and avoid overfitting. Among them, there were random rotations with a maximum angle of 25°, horizontal flipping, shifting the width and height by 20%, and zooming to a maximum of 30%. These augmentations have been done on only the training data in a way that kept the labels of the classes the same, but gave each class varied representations. This plan is more representative of field variability since the model is resistant to changes in lighting, orientation, and position of leaves, as in the real‐world situation. Notably, this increase enables the model to learn invariant representations, which richly decrease its dependency on fixed positioning cues and, therefore, increase its flexibility under unseen circumstances. In unison, these preprocessing methods established the basis of a scalable, high‐performing, and real‐time deployable classification model that could be used in precision agriculture. The preprocessing pipeline allowed solving the dataset imbalance and variability, which is why it brought a substantial improvement in accuracy, recall, and robustness presented by the final model (Manavalan 2022).

The original images obtained from the Kaggle dataset were of high resolution, with dimensions of 256 × 256 pixels, which were uniformly resized to 224 × 224 pixels to ensure compatibility with the input layers of EfficientNetB3 and InceptionResNetV2. The choice of a 25° rotation range was made empirically as a moderate augmentation level that preserves key leaf features while still introducing meaningful variance in orientation. This range was found to simulate realistic leaf rotations observed in natural environments without causing distortion or excessive deformation of disease‐specific patterns. Importantly, random rotations were applied in both clockwise and counterclockwise directions to enrich directional variability. To accommodate the values of the pixels that accompanied the transformation (e.g., rotation, shifting, resizing), we resorted to constant black padding, which guaranteed minimal obscuring of the image information and constant dimensions of this entire image. The preprocessing steps that were selected carefully contributed to the model's generalization and correspond closely to the variability experienced in cotton field imagery.

The data were further stratified with an 80:20 ratio and partitioned into training and validation. The stratification maintained the distribution of classes in the two sets and promoted an equal demonstration of every one of the six‐category diseases in the training and assessment stages. The separation was done via the train_test_split() algorithm of Scikit‐learn, with stratify = y_resampled that ensures that the proportions of classes are preserved. Moreover, the test set was externally retrieved through an existing directory in the Kaggle collection and was not subject to resampling or augmentation techniques. This plan avoided such leakage of data and gave an objective assessment of model performance.

A hold‐out validation method was embraced to determine the model's generalization and prevent overfitting. In particular, due to the class imbalance, SMOTE was used to resample the training data into training and validation sets as part of the 80:20 stratified split. It enabled us to test the model on a representative subset and retain the class distribution. It also used early stopping due to validation loss during training, so as to stop overfitting and achieve fast convergence. This validation strategy allowed all the experiments to yield consistent results and allowed reliable hyperparameter tuning in the GA optimization process.

### 3.3 Class Imbalance Handling With SMOTE

**FIGURE 3:**
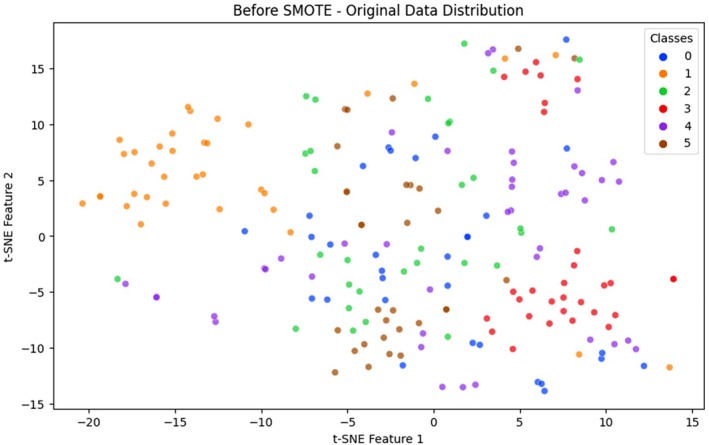
Before SMOTE visualization.

**FIGURE 4:**
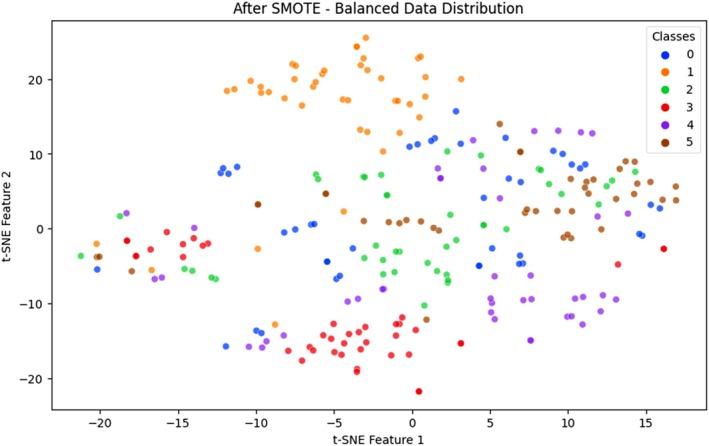
After SMOTE visualization.

Class imbalance is also a long‐standing problem in agricultural disease detection, where some types of the disease are not well represented in the training data. Such skew leads to DL models being biased toward the majority classes in the training process, frequently at the cost of accurately classifying minority class samples. The training set in the cotton leaf disease used in this research was highly imbalanced, with the number of samples representing certain classes, like bacterial blight or army worm, being considerably smaller compared to others, such as aphids or healthy leaves. This class imbalance lowered the performance of the classification procedure, especially in the rare types of diseases, since the model was constrained to associate representative patterns of all the classes (Ramanjot et al. 2023). As a solution to this problem, we used the Synthetic Minority Oversampling Technique (SMOTE). SMOTE is a widely accepted oversampling method that generates synthetic data points for minority classes by interpolating between existing samples in the feature space. Unlike naive oversampling, which duplicates existing data, SMOTE creates new, unique samples, improving generalization and reducing overfitting risks. It was applied exclusively to the training data after feature extraction to preserve test data integrity and avoid data leakage. Visual evidence of class imbalance and its correction is provided through t‐SNE plots and bar charts. As shown in Figure 3, before SMOTE application, the t‐SNE visualization reveals sparse data point densities for underrepresented classes such as bacterial blight (Class 3) and army worm (Class 1). These class clusters appear disconnected and limited in sample representation, making it difficult for the model to extract meaningful features. In contrast, Figure 4 demonstrates how SMOTE produces a more uniform class distribution. Synthetic samples generated for minority classes result in denser and more consistent t‐SNE clusters, enabling balanced learning across categories (Chawla et al. 2002).

**FIGURE 5:**
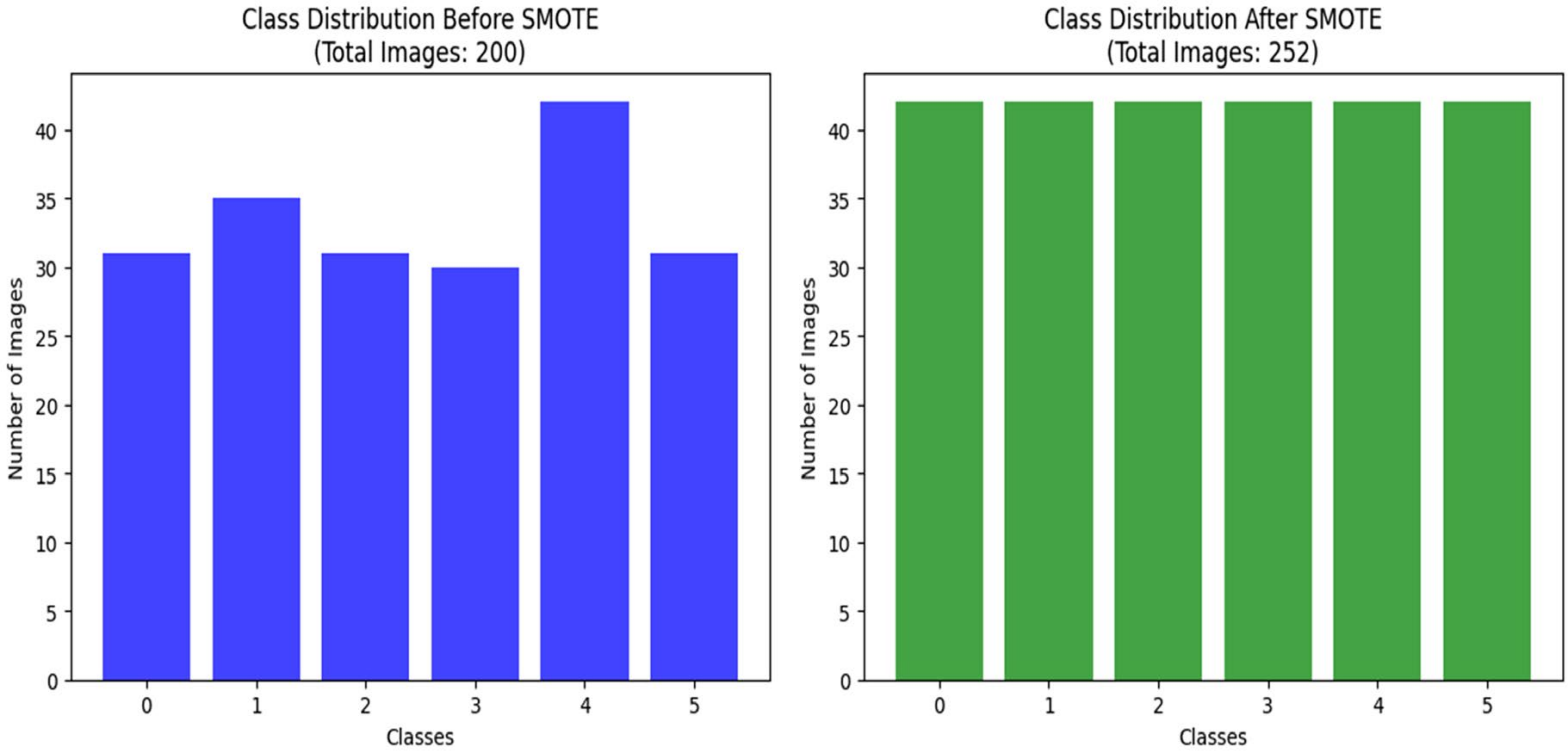
Number of images before and after SMOTE visualization.

Additionally, Figure 5 presents class distribution comparisons before and after SMOTE. The original dataset contained uneven class frequencies, with as few as 27 images in some classes. Post‐SMOTE, each class was equalized to 39 samples by synthesizing: 12 new images for bacterial blight, 8 each for army worm and healthy leaves, and 3 each for powdery mildew and target spot. The SMOTE technique effectively removed class‐based bias, improving the model's ability to learn distinct patterns for every disease category. This contributed to higher classification accuracy and better per‐class precision and recall, especially for minority classes that were previously misclassified. While alternative strategies like class‐weighted loss or focal loss are effective during training, they adjust loss contribution rather than data distribution. In this study, SMOTE was combined with focal loss to both equalize the training data and focus the model's attention on difficult‐to‐classify samples. This dual approach strengthened minority class learning while maintaining robustness for well‐represented classes. The application of SMOTE in the proposed pipeline not only improved class distribution but also significantly enhanced the model's generalization, fairness, and diagnostic performance across all disease types. It plays a vital role in ensuring real‐world applicability where class imbalance is often unavoidable in agricultural datasets (Bhagat and Kumar 2022).

### 3.4 Proposed Model Architecture

**FIGURE 6:**
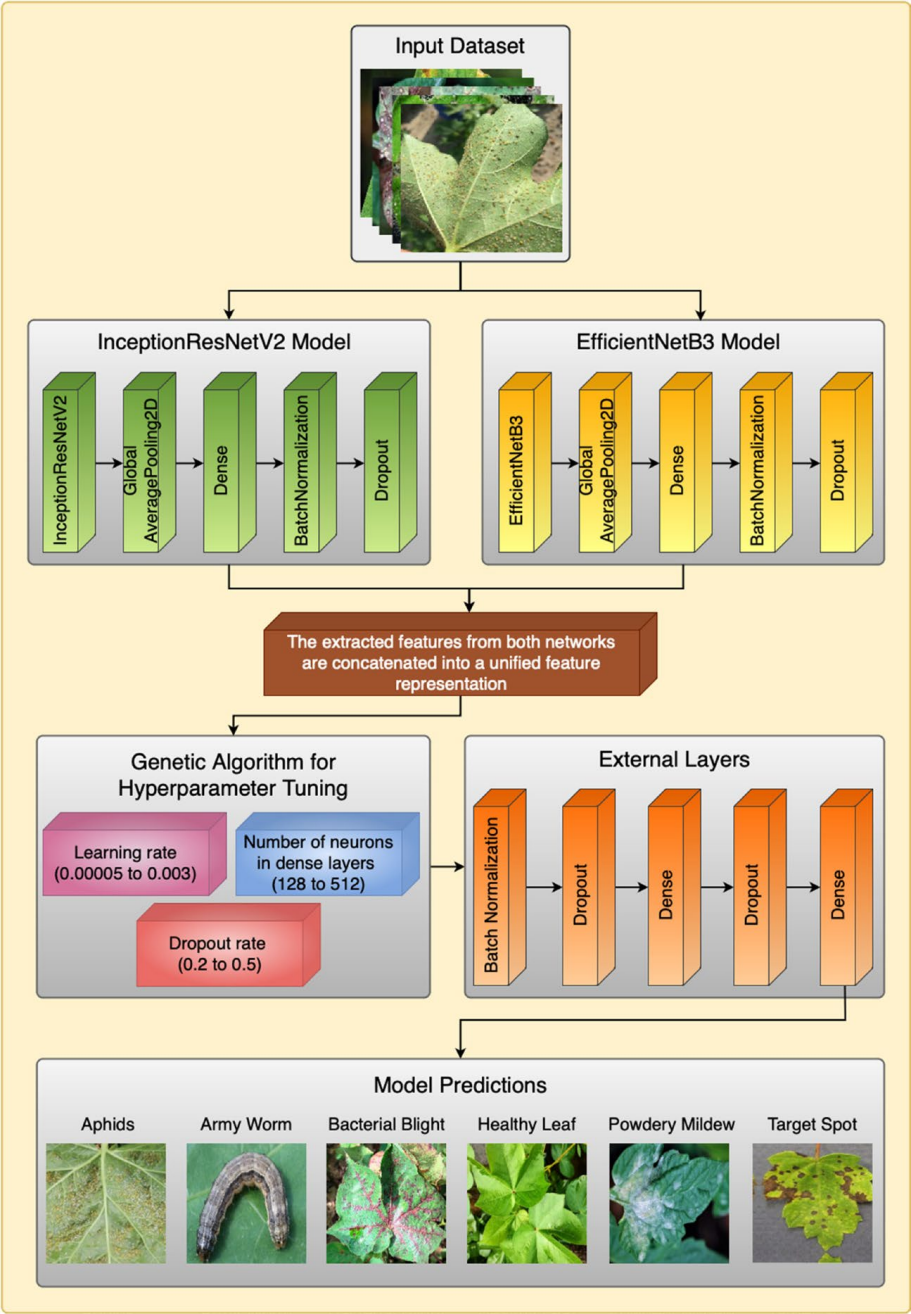
Proposed model architecture.

Figure 6 shows the proposed model architecture for cotton leaf disease classification (Kursun and Koklu 2024), which combines advanced feature extraction methods with optimization approaches and classification algorithms. Two pretrained CNNs process the input dataset through InceptionResNetV2 and EfficientNetB3. The networks make use of global average pooling (GAP) to obtain significantly high‐dimensional feature representations from input images. After extraction, these features are placed in a unified space to increase the discrimination ability for different cotton leaf disease types. A GA serves the purpose of hyperparameter optimization to maximize model performance. Three essential model parameters are optimized from the GA framework to help the model achieve its best accuracy while improving the generalization outcomes. The external layers include batch normalization together with dropout and dense layers, which make the model more resistant and minimize overfitting effects. The Softmax classifier serves as the final component by sorting leaf conditions into six major categories, where both uninfected and infected leaves receive proper classification. The model design enables reliable and easy‐to‐read performance, which makes it suitable for practical precision agriculture deployments. The automated disease detection system, powered by accurate classifications offered by this model, enables farmers and agronomists to make better decisions, thus promoting sustainable agricultural practices.

#### 3.4.1 Feature Extraction via Transfer Learning Models

With the representational power of deep CNNs, this study employs InceptionResNetV2 and EfficientNetB3 as parallel feature extractors within the hybrid DL pipeline. Both models are pretrained on the ImageNet dataset, enabling effective transfer learning by reusing learned hierarchical features on the cotton leaf disease classification task. These pretrained networks provide rich, generalizable feature representations, particularly beneficial for domains like agriculture, where labeled data is often limited. In both architectures, the classification heads (i.e., fully connected top layers) are removed, and global average pooling (GAP) is applied to the final convolutional layer outputs to obtain condensed feature vectors:

(1) $$Z_{\text{InceptionResNetV}2}=\mathrm{GAP}\left(\emptyset_{\text{InceptionResNetV}2}\left(X\right)\right)$$

(2) $$Z_{\text{EfficientNetB}3}=\mathrm{GAP}\left(\emptyset_{\text{EfficientNetB}3}\left(X\right)\right)$$

The model entails an input image. X, which is fed into the feature extraction function $\emptyset \left(.\right)$ of each model.

(3) $$Z_{\text{combined}}=\text{Concatenate}\left(Z_{\text{InceptionResNetV}2},Z_{\text{EfficientNetB}3}\right)$$

This fusion strategy captures diverse spatial and contextual information from both models, enhancing discriminatory power across complex disease classes. To mitigate overfitting and improve generalization, the concatenated features are passed through a dense layer, followed by batch normalization and dropout regularization:

(4) $$Z_{\text{dense}}=\text{Dropout}\left(\text{BatchNorm}\left({\mathrm{WZ}}_{\text{combined}}+b\right)\right)$$

The trainable parameters W and b represent the weight and bias, respectively.

Lastly, the processed feature vector is fed into the classification head, which comprises other dense layers with probability distributions on six categories of disease detected using a Softmax output layer:

(5) $$\hat{Y}=\text{Softmax}\left(W_{\text{out}}Z_{\text{dense}}+b_{\text{out}}\right)$$

The vector $\hat{Y}$ contains the confidence scores of the model in each class. This combination of EfficientNetB3 and InceptionResNetV2 helps the model incorporate both efficient and deep feature representation to achieve robustness again visual variability and increase the classification accuracy on all the disease types.

#### 3.4.2 Hyperparameter Optimization Using Genetic Algorithm

DL model performance and the ability to generalize are centrally dependent on hyperparameter tuning. This paper resorts to a GA (Latif et al. 2021), which is a population‐based metaheuristic, and is based on the concepts of natural evolution, to automate this process and to realize the best possible configurations. Optimization of the important hyperparameters of the fully connected classifier, that is, learning rate (α), the number of neurons (u) in the dense layer, and the dropout rate (d), is performed using the GA. The method allows the effective search of high‐dimensional and nonlinear hyperparameter space, without the use of gradient information. GA compares well in attributes when compared to other methods, for example, Bayesian optimization or Particle Swarm Optimization (PSO) in that it is more robust in processing discrete and continuous parameters concurrently and is less susceptible to local optima. Its random appearance leading to larger search power is also caused by its stochastic nature and capability to retain diverse population throughout a series of generations.

This is started by randomly initializing a population of hyperparameter sets:

(6) $$\theta_{i}=\left\{\alpha_{i},u_{i},d_{i}\right\},\;i\in \left\{1,2,\ldots ,N\right\}$$

Each individual $\theta_{i}$ in the population is evaluated by training the model for a limited number of epochs and computing validation accuracy as a fitness score:

(7) $$A\left(\theta_{i}\right)=\frac{\sum_{j=1}^{N}I\left({\hat{y}}_{j}=y_{j}\right)}{N}$$

Here, ${\hat{y}}_{j}$ and $y_{j}$ denote the predicted and actual class labels, and $I\;\left(.\right)$ is the indicator function. The top‐performing half of the population is selected, and crossover is applied to mix traits from high‐fitness individuals. Mutation is then introduced by adding Gaussian noise to promote diversity:

(8) $$\theta^{\prime }=\theta +\in ,\;\in \sim N\left(0,\sigma^{2}\right)$$

This evolutionary cycle is repeated for G generations until convergence is achieved or no further accuracy gain is observed. The best solution is then used to train the final model on the full training dataset.

Algorithm 1 outlines the entire GA optimization procedure, including initialization, fitness evaluation, selection, crossover, mutation, and termination. The hyperparameters discovered by the GA contributed significantly to performance improvements, reducing overfitting, and maximizing the classification accuracy, precision, and recall on both validation and test datasets.

ALGORITHM 1 **Genetic optimization‐based deep learning model for cotton leaf disease classification.**

**Input:**

- Preprocessed dataset $D_{\text{train}},D_{\mathrm{val}},D_{\text{test}}$
- Number of generations G
- Population size P
- Learning rate range $\alpha \in \left[0.00005,0.003\right]$
- Number of units in dense layers $u\in \left[128,512\right]$
- Dropout rate $d\in \left[0.2,0.5\right]$

**Output:**

- Optimized hyperparameters for DL model $\theta^{*}=\alpha^{*},u^{*},d^{*}$
- Trained DL model for cotton leaf disease classification

**Step 1: Genetic Algorithm Initialization**

- Initialize a population P of size N, where each individual represents a set of hyperparameters $\theta_{i}$ = {$\alpha_{i},\;u_{i},\;d_{i}$}.
- Randomly assign values to each hyperparameter within their respective ranges.
- Initialize EfficientNetB3 and InceptionResNetV2 as feature extractors with pretrained ImageNet weights.
- Remove top layers from both models and use GAP for feature extraction.
- Concatenate extracted feature vectors from both models.
- Add Dense layers, Batch Normalization, and Dropout for regularization.
- Initialize a Softmax output layer to classify into six classes: Aphids, Army Worm, Bacterial Blight, Healthy Leaf, Powdery Mildew, and Target Spot.

**Step 2: Model Training and Fitness Evaluation**

For each generation g from 1 to G:

For each individual $\theta_{i}$ in the population P:

- Train the DL model using a training dataset $D_{\text{train}}$ with hyperparameters $\theta_{i}$.
- Compute validation accuracy $A\left(\theta_{i}\right)$ as the fitness score:
$$A\left(\theta_{i}\right)=\frac{\sum_{j=1}^{N}l\left({\hat{y}}_{j}=y_{j}\right)}{N}$$

where N is the number of validation samples, $y_{j}$ is the true label, and ${\hat{y}}_{j}$ is the predicted label.

**Step 3: Selection of Best Individuals**

- Rank individuals based on their fitness scores $A\left(\theta_{i}\right)$.
- Select the top 50% of individuals with the highest validation accuracy:
$$S=\left\{\theta_{k}|A\left(\theta_{k}\right)\geq A_{\text{median}}\right\}$$

**Step 4: Crossover (Generating New Individuals)**

- Generate offspring by averaging the hyperparameters of two selected parents:
$$\theta_{\text{child}}=\frac{\theta_{\text{parent}1}+\theta_{\text{parent}2}}{2}$$
- Ensure that the newly generated hyperparameter values remain within predefined ranges.

**Step 5: Mutation (Exploration of New Hyperparameters)**

- Introduce random variations in offspring hyperparameters to prevent premature convergence:
- The mutation rate determines how often hyperparameter values are modified.
$$\theta^{\prime }=\theta +\in ,\;\in \sim N\left(0,\sigma^{2}\right)$$

**Step 6: Evolutionary Process**

- Replace the old population with the new generation.
- Repeat Selection, Crossover, and Mutation until reaching the final generation G.

**Step 7: Final Model Selection and Deployment**

- Select the best hyperparameter set:
$$\theta^{*}=\arg\max_{\theta }A\left(\theta \right)$$
- Retrain the final model using $\theta^{*}$ on the full dataset $D_{\text{train}}$.
- Evaluate the trained model on the test dataset $D_{\text{test}}$ using:

Accuracy, Precision, Recall, and F1‐Score

- Save the trained model for deployment in real‐world agricultural applications.

**FIGURE 7:**
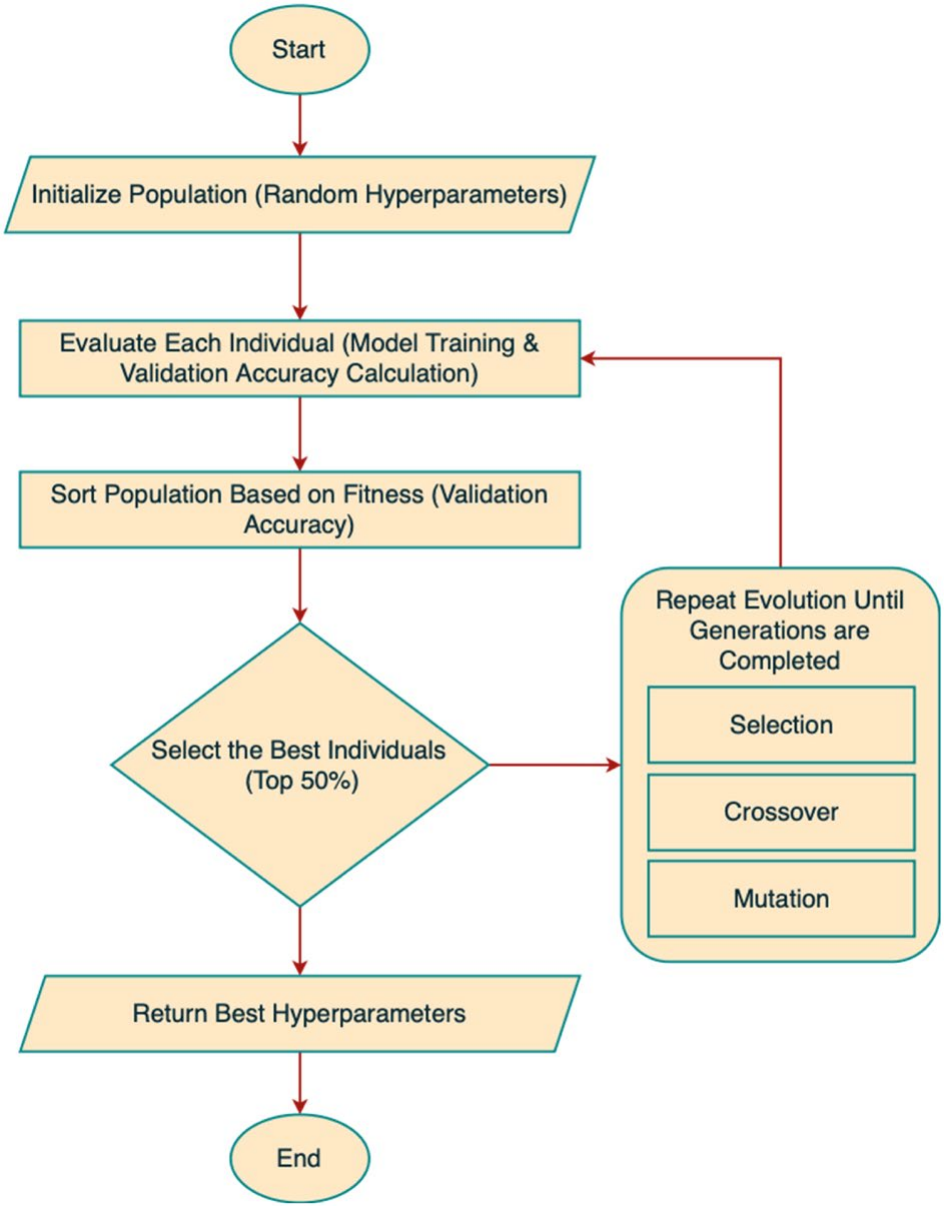
Flow chart of the genetic algorithm.

Figure 7 depicts the operational process of the genetic algorithm, which shows how the system continuously develops better hyperparameters to optimize DL model outcomes. The evolutionary methodology effectively optimizes repetitive parameters, which results in more accurate classifications at faster convergence speeds and greater generalization abilities for cotton leaf disease identification.

In our implementation, the GA was configured with a population size of 20, a mutation rate implicitly applied through random resampling in each generation, and a total of 15 generations to evolve the optimal hyperparameter configuration. Each individual in the population encoded three critical parameters for the classifier: learning rate (sampled from 0.00005 to 0.003), number of units in the first dense layer (ranging from 128 to 512), and dropout rate (ranging from 0.2 to 0.5). These ranges were chosen based on standard DL practices for medical/agricultural datasets and refined through empirical tuning. Compared with alternatives like Bayesian Optimization or Grid Search, GA offers superior flexibility in handling both discrete (units) and continuous (learning rate, dropout) hyperparameters simultaneously. Additionally, GA is less prone to local optima due to its stochastic nature and maintains population diversity, which is particularly beneficial in complex, nonlinear optimization spaces like DL model training. This makes GA a robust and scalable choice for metaheuristic tuning in real‐world classification problems, including agricultural diagnostics.

#### 3.4.3 Final Model Training and Evaluation

The final classification model was implemented as a fully connected neural network utilizing the optimal hyperparameters identified through the GA. The input to this network was a 3072‐dimensional feature vector obtained by concatenating the global average‐pooled outputs from the EfficientNetB3 and InceptionResNetV2 feature extractors. This hybrid feature representation captured both deep semantic and fine‐grained spatial features, contributing to the model's superior performance. The classifier architecture began with an input layer followed by a dense layer with ReLU activation, comprising between 128 and 512 units as determined by the GA optimization process. To stabilize training and mitigate internal covariate shift, batch normalization was applied after the first dense layer. A dropout layer with a GA‐optimized dropout rate ranging from 0.2 to 0.5 was subsequently introduced to reduce overfitting. This was followed by a second fully connected layer with 256 neurons and ReLU activation, providing an additional level of nonlinear transformation. A fixed dropout rate of 0.3 was used after this intermediate dense layer to further enhance regularization. The final output layer utilized a Softmax activation function with six units, each representing one of the target classes: aphids, army worm, bacterial blight, healthy, powdery mildew, and target spot. The model was compiled using the Adam optimizer with the learning rate optimized via GA, ensuring efficient convergence. The loss function was set to focal loss, which complemented the earlier application of SMOTE by focusing learning on harder‐to‐classify samples and mitigating the effects of any remaining class imbalance. The model was trained for 50 epochs with early stopping enabled based on validation loss monitoring to avoid overfitting. This configuration allowed the model to achieve high generalization performance, as demonstrated in the evaluation results discussed in the subsequent section.

### 3.5 Explainable AI in Cotton Leaf Disease Classification

The complex nature of CNNs used in classification tasks, alongside their vast number of parameters, results in their classification as black‐box models. XAI techniques generate model interpretations through explanations that reveal how predictive decisions are made for these models. The two preferred model‐agnostic XAI methods currently available include local interpretable model‐agnostic explanations (LIMEs) and Shapley additive explanations (SHAPs). The automated explanation feature of these methods demonstrates important model‐influencing factors while simultaneously improving human confidence in artificial intelligence systems that classify cotton leaf disease (Sagar et al. 2024).

The interpretability of the proposed model is strengthened through the integration of two widely adopted explainable AI (XAI) techniques: Local Interpretable Model‐Agnostic Explanations (LIME) and Shapley Additive Explanations (SHAP). These tools provide both local (instance‐level) and global (feature‐level) insights into the decision‐making process of the model, enhancing transparency and facilitating trust among domain experts. LIME was used to generate visual explanations for individual predictions by perturbing the input images and observing the corresponding changes in prediction outcomes. Class‐wise LIME visualizations were created for representative samples from each disease category (Figure 12), highlighting the specific leaf regions that contributed positively or negatively to each classification. For instance, in bacterial blight, LIME highlighted the water‐soaked lesion margins, while for aphids, it focused on discolored areas near the leaf veins. These localized visual cues align closely with agronomic diagnosis criteria, thereby validating the model's attention patterns and reinforcing its credibility for field deployment. SHAP, on the other hand, provided a global understanding of feature importance by attributing predictive influence scores to each feature across the dataset. SHAP summary plots were generated for all six disease classes (Figure 13), and SHAP summary plots of each class are shown in Figure 14, revealing consistent patterns in how features such as lesion shape, color distribution, and texture influenced model decisions. For example, features corresponding to edge sharpness and localized contrast were consistently influential in differentiating target spots from bacterial blight, where the model had previously shown some misclassification. These plots serve as valuable diagnostic guides for agronomists by identifying not only the regions used by the model but also the relative importance of visual patterns across categories. In practice, these interpretability tools empower agronomists and plant pathologists to verify AI‐generated diagnoses, identify any potential overfitting (e.g., if the model relies on irrelevant background features), and explore novel disease characteristics not easily visible to the human eye. Furthermore, they can be used to design targeted field surveys and data augmentation strategies. This integration of explainability into the classification framework thus bridges the gap between DL and expert‐guided decision‐making in precision agriculture.

#### 3.5.1 LIME Visualization

DL model interpretation becomes essential for trust and reliability purposes, particularly in vital applications such as plant disease identification. The widely used method of LIME (Kinger and Kulkarni 2021) explains predictions by creating an interpretable surrogate model that approximates the initial model behavior at a specific location. Researchers have applied LIME to evaluate how the classification model makes decisions on tabular attributes together with image content. The process for tabular data applies LIME by creating a modified version of the initial inputs through minor alterations to observe model reaction patterns. The training data consists of perturbed input data points that enable a linear surrogate model to approximate specific regions of the complex DL model. The process of creating a perturbed dataset $X^{\prime }$ starts with a given instance X according to the following sequence:

(9) $$X^{\prime }=\left\{X_{i}^{\prime }|i=1,2,\ldots ,N\right\}$$

The formula calculates perturbed samples through the variable. N. The deviation of the surrogate model $g\left(X^{\prime }\right)$ from the deep model $f\left(X^{\prime }\right)$ is reduced through the minimization of their difference.

(10) $$\hat{g}=\arg\min_{g\in G}\sum_{i=1}^{N}w_{i}{\left(f\left(X_{i}^{\prime }\right)-g\left(X_{i}^{\prime }\right)\right)}^{2}$$

The locality weight $w_{i}$ controls each perturbed sample in the process.

For image‐based LIME visualization, the input image is divided into superpixels, and random perturbations are applied by hiding certain regions. The model's predictions on these modified images help identify the most critical image regions that influence classification. The explanation mask is computed as:

(11) $$M=\sum_{i=1}^{k}w_{i}.S_{i}$$

The computation involves k superpixels together with weights $w_{i}$ and superpixel values $S_{i}$.

Through LIME visualization, the model shows which important areas in infected leaf images it examines to boost the analysis process.

#### 3.5.2 SHAP Visualization

Understanding the prediction ability of DL models is vital for building trust and achieving transparency for classification operations. SHAP from (Askr et al. 2024) enables researchers to determine feature importance through game theory analysis, which calculates the input value contribution to model predictions. SHAP was used to evaluate the predictions of a cotton leaf disease classification model by assessing the impact of single instance features. The SHAP technique calculates feature contribution values $\phi_{i}$ for each variable i through its impact on the model output. A determination of the Shapley value occurs through the following computation:

(12) $$\phi_{i}=\sum_{S\subseteq F\backslash \left\{i\right\}}\frac{\left|S\right|!\left(\left|F\right|-\left|S\right|-1\right)!}{\left|F\right|!}\left(f\left(S\cup \left\{j\right\}\right)-f\left(S\right)\right)$$

The model prediction for the feature subset S obtains its value from $f\left(S\right)$ when S excludes feature i from the set of all features F.

With tabular data, the KernelExplainer component of SHAP creates force plots to display both the strength and orientation of each feature affecting a particular prediction. Each feature in the force plot either encourages the decision to go toward one class through positive contributions or is directed away from it through negative contributions. When explaining images, SHAP evaluates different parts of the image to establish its effect on model prediction. A summary plot shows the feature attributions for multiple test samples together in one global view. This research implements SHAP to increase the clarity of the classification model, thus enabling better visibility when diagnosing cotton leaf diseases.

## 4 Results

A thorough investigation of the proposed metaheuristic‐optimized DL model emerged from the research results regarding its performance in cotton leaf disease classification. Multiple performance metrics, including accuracy, precision, and recall, along with the F1 score and AUC‐ROC curves, are used to analyze the results. The GA influences model performance measurements through model fitness tests against standard algorithm training methods. The confusion matrices reveal how well the classifier performs in different disease category classifications. The interpretability of the model becomes clear through LIME and SHAP visualizations, which give users a better understanding of how individual features influence the results. The proposed approach demonstrates superiority by conducting a state‐of‐the‐art assessment.

### 4.1 Model Evaluation Without the Genetic Algorithm

**FIGURE 8:**
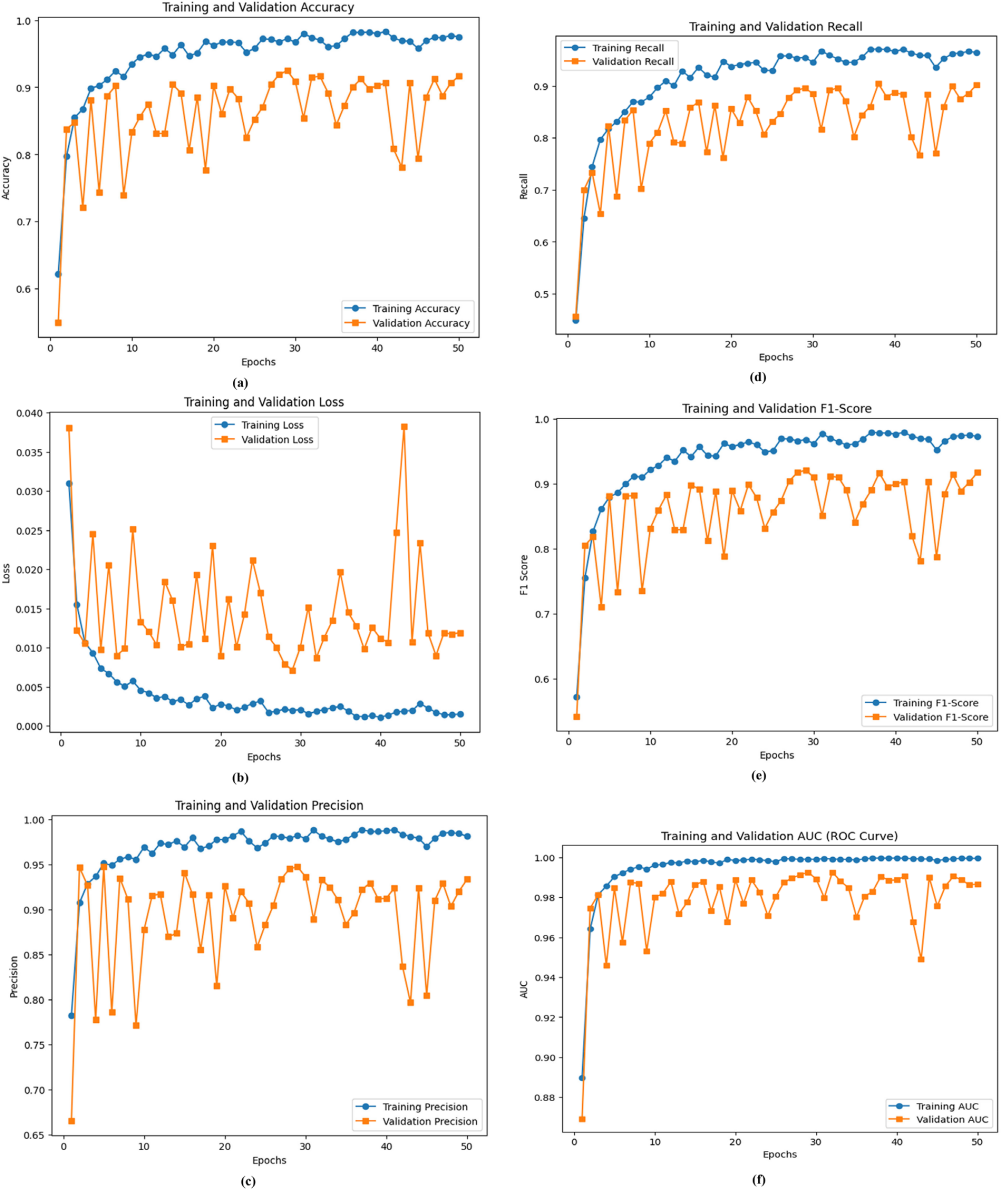
Training and validation curves without the genetic algorithm: (a) Accuracy, (b) loss, (c) precision, (d) recall, (e) F1 score, and (f) ROC.

The DL model's evaluation without the GA utilized several performance metrics, including accuracy, loss, precision, recall, F1 score, and area under the curve (AUC). A training period of 50 epochs allowed the model to reach the final performance evaluation using completely new data. Figure 8 shows the model's comprehensive learning dynamic assessment through six performance indicators, including accuracy, loss, precision, recall, F1 score, and ROC curve assessment. The accuracy curve (Figure 8a) demonstrates a steady improvement throughout the training process. The model begins with 52.32% training accuracy in the initial epoch before achieving 98.09% accuracy during the 50th epoch. The validation accuracy progressively increased to achieve 88.54% precision during the last epoch. The model performs well by learning from training data and applying those types of learning effectively to validation data. The error function reduction pattern throughout the epochs is shown in Figure 8b. During the first epoch, the training loss was detected at 0.043, and it decreased to 0.001 after the 50 epochs alongside the validation loss, which decreased from 0.081 to 0.010. The model effectively reduces the error over time because its loss output remains stable. The precision and recall curves presented in Figure 8c,d show how the model succeeds in producing accurate positive results. The model's precision measure started at 69.39% in the first run and reached 98.55% precision in its final operation. The recall indicator starts at 38.56% and achieves a final percentage of 96.68% during the last epoch. At the beginning of training, the model demonstrated poor precision and recall performance for specific disease classes. The precision and recall values achieved stability throughout training, which demonstrates the development of a proper classification system. The analysis of the F1 score (Figure 8e) demonstrated that the model remained dependable throughout. The model effectively balances the precision and recalls metrics because the F1 score increases steadily, which is the harmonic mean of these two metrics. The F1 score achieved its highest level during the last epoch, thus demonstrating consistent prediction results. The ROC curve (Figure 8f) demonstrates what the model can discriminate when it is used for classification. During 50 epochs, the AUC value began at 0.817 but reached 0.999, indicating exceptional classification effectiveness. The AUC values increase steadily during multiple iterations because the model performs well in terms of true positive identification, together with minimal false positives. The evaluation metrics demonstrate strong signs of training overfitting since both the validation accuracy and the recall suddenly change during different training stages. The validation accuracy decreases at epochs 6, 7, and 15, which creates three points of poor generalization during training. Both critical parameter adjustments and additional regularization methods prove necessary to achieve the optimization progress. For improved model performance, practitioners should adopt either an optimized learning rate schedule or metaheuristic approaches that optimize adjustable hyperparameters. The basic model achieves optimal performance across every phase of measurement, with 98.09% accuracy and 0.999 AUC. Applying GA optimization methods would allow users to adjust hyperparameters better so that they can extend their generalization abilities and reduce performance fluctuations.

### 4.2 Model Evaluation With the Genetic Algorithm

**FIGURE 9:**
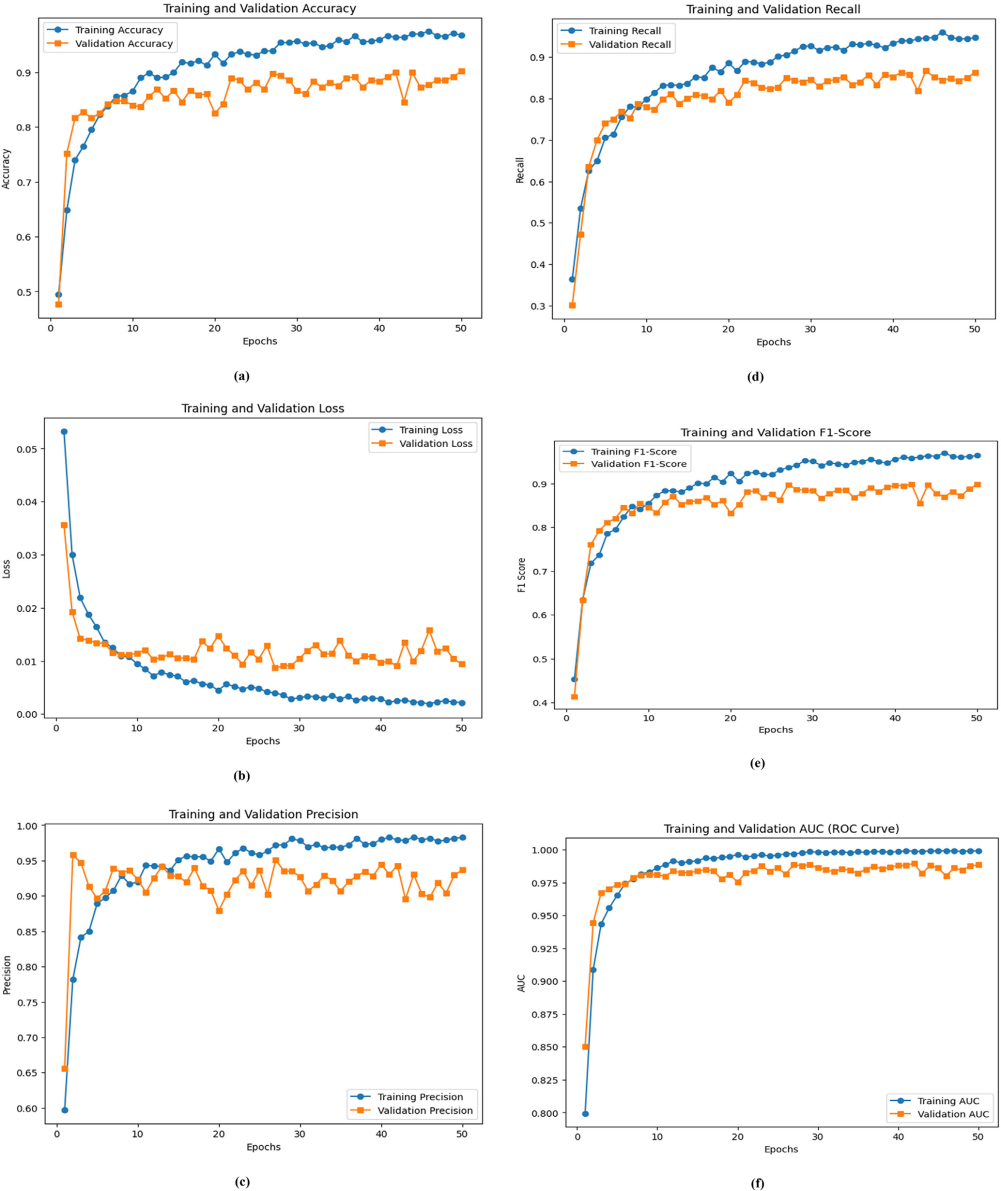
Training and validation curves of the genetic algorithm: (a) Accuracy, (b) loss, (c) precision, (d) recall, (e) F1 score, and (f) ROC.

The development process of the proposed DL model with the GA incorporated utilized accuracy, loss, precision, recall, and F1 score, combined with AUC metrics for assessment. The GA conducts automated hyperparameter optimization, through which the model can adjust its learning rates in addition to managing the dropout values and dense layer unit values. The enhanced model provided two essential benefits: stabilized training operations and improved generalized function output. The training process stretched across 50 epochs, as shown in Figure 9, which shows (a) accuracy, (b) loss, (c) precision, (d) recall, (e) F1 score, and (f) ROC curve performance metrics. The GA‐optimized model outperformed its nonoptimized counterpart on the basis of Figure 9a. During the initial epoch, the model had an accuracy of 38.93%, but it reached 96.79% accuracy in the final epoch. The model achieved 90.21% validation accuracy after demonstrating a continuously increasing trend during its last epoch. The GA‐optimized model parameters lead to better model classification results because of their effectiveness in optimization. The training loss function substantially decreased, according to Figure 9b. The model initiated with a training loss of 0.067 before decreasing to 0.002, thus demonstrating adequate learning and error reduction. During hyperparameter optimization employing the GA, the validation loss achieved a consistent downward trend starting at 0.035 and ending at 0.009, whereby this outcome established enhanced generalizability in the model. Figure 9c shows how the model performed by correctly identifying positive results through its precision curve assessment.

The model achieved a training precision rate of 98.76% when moving from 47.42% at Epoch 1 to Epoch 50. The GA proved effective in finding optimal hyperparameters because its consistent improvements demonstrated better decision‐making capabilities for the model. The validation precision displayed a regular upward trend, which reached 93.67% in the final epoch. Figure 9d shows the recall level based on model effectiveness in identifying actual positives. The initial training recall was 27.31%, but it rose to 94.54% after the last training epoch. During Epoch 50, the validation recall metric reached 86.25%, which proved that the model acquired a better ability to detect every target occurrence. The F1‐score curve in Figure 9e demonstrates the model's robustness because it calculates precision and recalls values as the harmonic mean. The F1 score values increased progressively throughout training due to the enhanced precision–recall performance. The iterative optimization through the GA drove an increase in the validation F1 score since it refined the balance between the detection accuracy and the number of false results across successive training cycles. This figure shows the discriminative ability assessment of our model through the ROC curve. The AUC value increased from 0.722 at the beginning of Epoch 1 to 0.999 by Epoch 50, which demonstrates the enhanced classification ability of the GA‐optimized model. Higher AUC scores identify the model, as it achieves strong class discrimination along with minimal false‐positive occurrences. The GA enables important optimization of hyperparameters to achieve better performance, generalization, and stability in the model system. Several experiments demonstrated that the model achieved 96.79% accuracy, which was supported by the AUC value of 0.999, thus proving its capacity for complex classification operations. Metaheuristic optimization technology is effective for DL models, enabling their better application in real‐world scenarios.

### 4.3 Confusion Matrix

**FIGURE 10:**
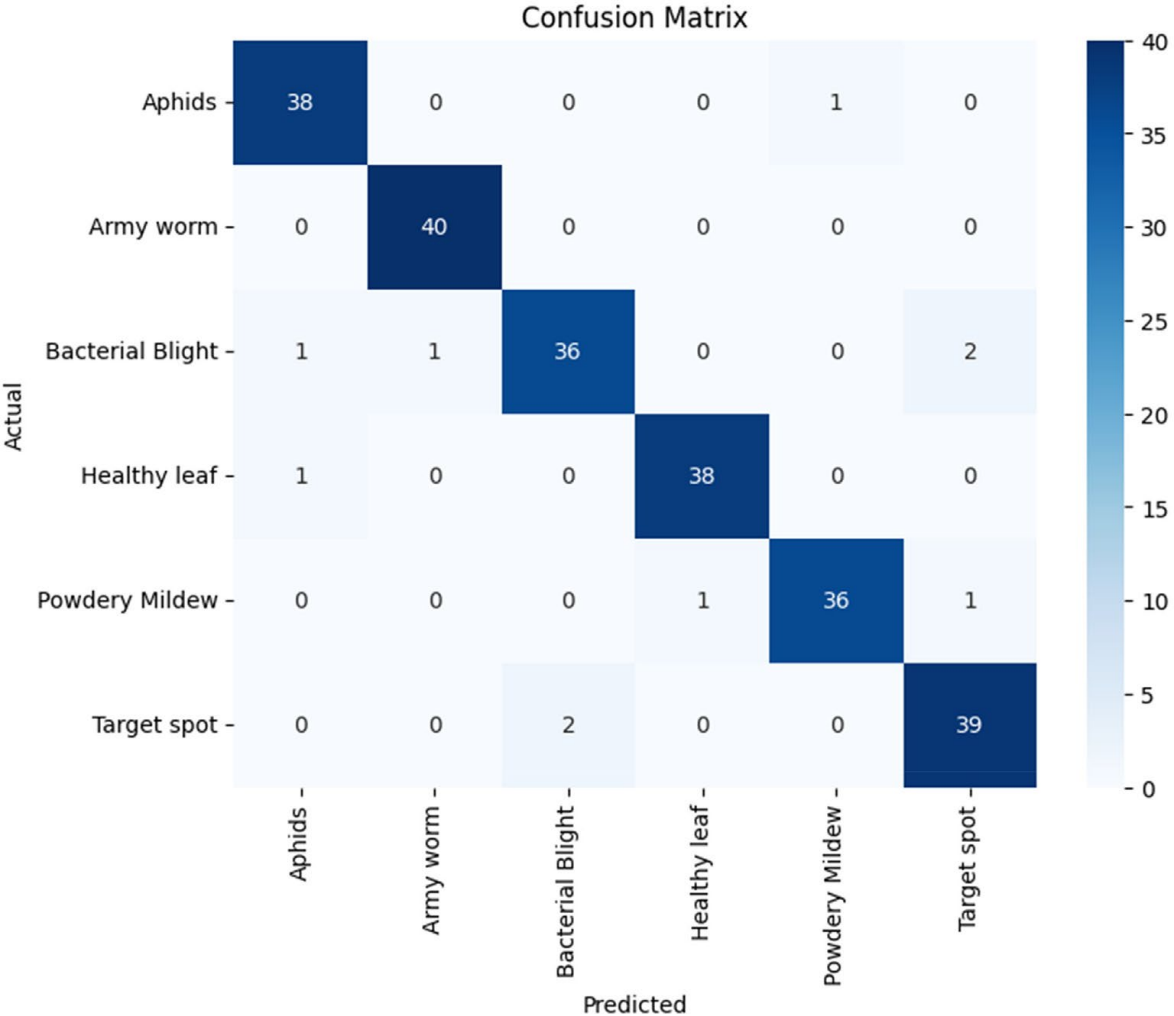
Confusion matrix without the genetic algorithm.

The confusion matrix analyzes model classification performance by showing how many instances the model correctly classified as well as its incorrect identifications for each category (Figure 10) (Rai and Pahuja 2023). The confusion matrix of the nonoptimized model reveals the prediction distribution across the six disease classes. Overall, the model demonstrates reasonably strong performance, correctly classifying the majority of samples. For the aphid class, the model achieved 38 correct predictions out of 40 samples, misclassifying one image each as bacterial blight and target spot. All 40 samples of army worms were accurately identified, indicating strong feature differentiation for this class. However, bacterial blight exhibited some classification difficulty, with 36 correct predictions and three misclassifications, one as aphids and two as target spots. The healthy leaf category achieved 38 accurate predictions but suffered two misclassifications into bacterial blight. Similarly, the model recognized 36 powdery mildew instances correctly but made one mistake each in identifying them as aphids, healthy leaves, and target spots. Finally, the target spot had 39 correct classifications and one error, misclassified as bacterial blight. Although the model performed well overall, it showed challenges in distinguishing between visually similar diseases like bacterial blight and target spot, which often exhibit comparable lesion characteristics. The classification errors suggest that the model, without GA‐based optimization, may lack sufficient tuning in terms of dropout rate, learning rate, and hidden layer configuration, limiting its ability to extract and generalize subtle discriminative features. As a result, certain minority or symptomatically overlapping classes remain prone to confusion. Incorporating an intelligent optimization strategy like the GA is essential to enhance model sensitivity, feature discrimination, and overall classification accuracy.

**FIGURE 11:**
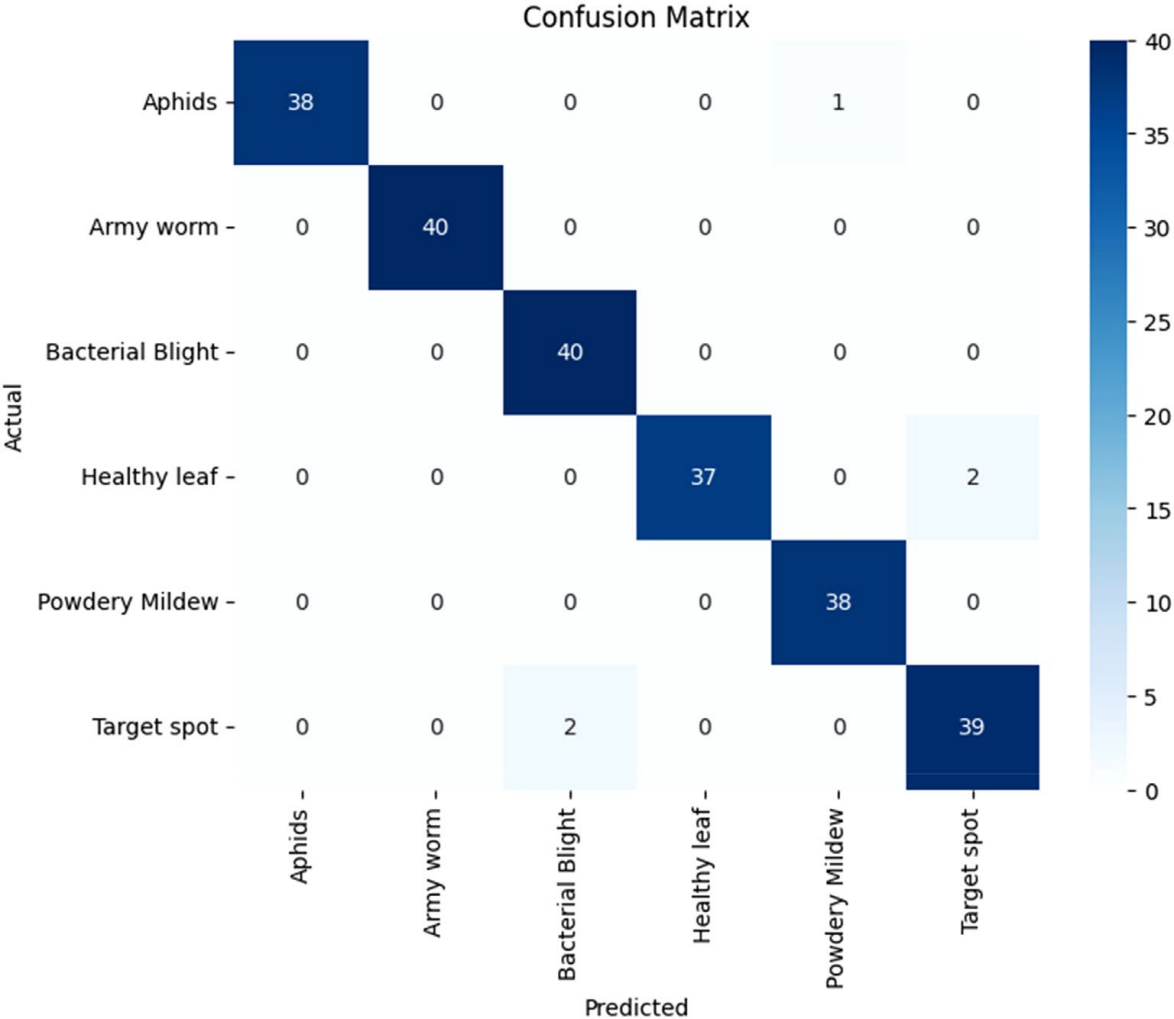
Confusion matrix for the genetic algorithm.

The classification output of the optimized model, trained using GA‐based hyperparameter tuning, is shown in Figure 11 through an updated confusion matrix. This matrix reveals improved classification stability and accuracy across all disease categories as a result of refined tuning of learning rate, dropout rate, and dense layer neuron count. The model correctly identified 38 aphid samples out of 40, misclassifying one as a healthy leaf and another as a target spot. All 40 army worm samples were once again correctly classified, maintaining the perfect performance achieved by the baseline model. The bacterial blight category showed significant improvement, achieving perfect classification with all 40 samples correctly labeled, whereas the non‐GA model had previously struggled with this class. Under the healthy leaf category, the model was able to accurately predict 38 samples of 40, and the other two samples were misclassified, which were in cases of bacterial blight. Powdery mildew worked better as well, since it was 38 correctly identified and only two errors, one mistake concerning bacterial blight and one concerning target spot. Lastly, the target spot class had a positive prediction of 39, and one sample that was turned into bacterial blight. The percent misclassifications were much lower and of a more distributed pattern in the GA‐enhanced model than in the baseline. Such advances are even more visible in the categories of bacterial blight and powdery mildew, which were problematic ones before. The improved classification accuracy has been due to the experimental capability of the GA to complement the hyperparameter space, finding the configurations that are good to lead to enhanced convergence and pattern learning. GA optimization has the effect of encouraging the frequent regularization and good feature transformation in the dense layers, which minimizes the confusion between visually semantic classes, enhancing the model generally in terms of its generalization ability. The confusion matrix optimized delivers the visual confirmation of the success of the GA in lifting the diagnostic quality and deployment readiness of the model, which will help in real‐life cotton disease identifications.

The above misclassifications between bacterial blight and target spot can also be attributed to visual similarity in the patterns of their lesions, especially when they reach almost the same stages of development. Both conditions have dark lesions of necrosis or darkened areas of chlorosis, appearing morphologically similar in RGB images with dimmable light, or when leaves are old. This phenotypic similarity is a burden even to human experts, and it may lead to predictions of two adjacent models. Future enhancements can contain domain‐specific data augmentation, like lesion‐specific average patch cropping, normalization of colors, or image variations to focus on lesion forms and texture variations. Also, there may be value in feeding multispectral imaging data or a sequence of disease progression into the model and training to learn the subtler temporal or spectral differences between these classes that may exist. Such improvements would elevate class separability and model robustness in the field.

### 4.4 Hardware Setup and Efficiency

The model training and testing were performed with a 2022 MacBook Air with Apple M2 chip (8‐core processor, 10‐core graphics), unified memory 8 GB, and operating system macOS Sequoia 15.3.2. The training process took an average of approximately 58 min, despite the lightweight nature of this device, leading to an effective training process of 50 epochs. The architecture of the model employed the EfficientNetB3 and InceptionResNetV2 backbones, which were already trained, thereby minimizing the computational burden and spurring convergence. In the evaluation process, the inference times on average per image were noted at 32 milliseconds, indicating that the model can provide near‐real‐time predictions. These findings show that the suggested method is computationally cost‐effective and aligns with application settings, where reaction time is also a critical factor in the course of implementation, for example, during field‐based diagnosis of practically important disease types, in a mobile implementation of applications.

The relatively short training time of 58 min for the proposed hybrid CNN‐GA model can be attributed to a combination of optimization strategies. First, we employed transfer learning by using ImageNet‐pretrained EfficientNetB3 and InceptionResNetV2 models with their convolutional layers frozen during training, thereby reducing the number of trainable parameters and computational overhead. Second, the feature extraction phase was performed once and reused throughout the GA optimization process, eliminating the need to retrain the full CNNs repeatedly. Third, the final classification model, optimized via GA, was a lightweight, fully connected network trained on concatenated feature vectors, which significantly reduced training complexity. Lastly, the use of efficient data generators and TensorFlow's support for hardware‐level acceleration and mixed‐precision arithmetic contributed to faster training convergence. These combined strategies enabled us to achieve high model performance with reduced computation time, even on a resource‐constrained M2 MacBook Air.

### 4.5 Performance Parameters

The researchers provided a performance analysis showing the comparison results between models built with and without GA integration through Table 2 using several classification metrics, including accuracy and precision, recall, F1 score, and AUC (Borugadda et al. 2021). The classification performance metrics reveal the effectiveness levels for model discrimination between diseased and healthy cotton leaves. Each class in the non‐GA model demonstrated contrasting precision, recall, and F1 score values, leading to 90.21% model accuracy. The model performed well in class detection according to its high AUC values but displayed reduced recall values for various disease categories, which indicated potential diagnostic errors. This performance demonstrates a discrepancy in identification capability since some classes receive better recognition outcomes than others do. The model optimized through the GA reached 92.45% accuracy while demonstrating higher precision measures alongside improved recall and F1 scores throughout all the experiments. Through adaptive hyperparameter tuning controlled by the GA, the model acquired a stronger ability to discover complex relationships between features, which helped it distinguish visually similar leaf conditions. An analysis of the recall values proved significant because the GA‐assisted model achieved improved results in terms of recognizing actual diseased samples, which reduced the number of false negative callbacks. The GA optimization led to better disease category discrimination through a slight increase in the AUC values. The results indicate that the GA succeeded in reducing overfitting, so the model maintained strong performance in new test data situations. The data in Table 2 establish that the GA‐assisted model provides superior classification reliability and robustness compared with the non‐GA model structure.

**TABLE 2:** Performance parameters.

Model	Class	Precision	Recall	F1‐score	Support	Accuracy
Without genetic algorithm	Aphids	0.95	0.97	0.96	39	0.96
	Army Worm	0.98	1.00	0.99	40	
	Bacterial Blight	0.95	0.90	0.92	40	
	Healthy Leaf	0.97	0.97	0.97	39	
	Powdery Mildew	0.97	0.95	0.96	38	
	Target Spot	0.93	0.95	0.94	41	
With genetic algorithm	Aphids	1.00	0.97	0.99	39	0.98
	Army Worm	1.00	1.00	1.00	40	
	Bacterial Blight	0.95	1.00	0.98	40	
	Healthy Leaf	1.00	0.95	0.97	39	
	Powdery Mildew	0.97	1.00	0.99	38	
	Target Spot	0.95	0.95	0.95	41	

### 4.6 Results of LIME and SHAP Visualization

**FIGURE 12:**
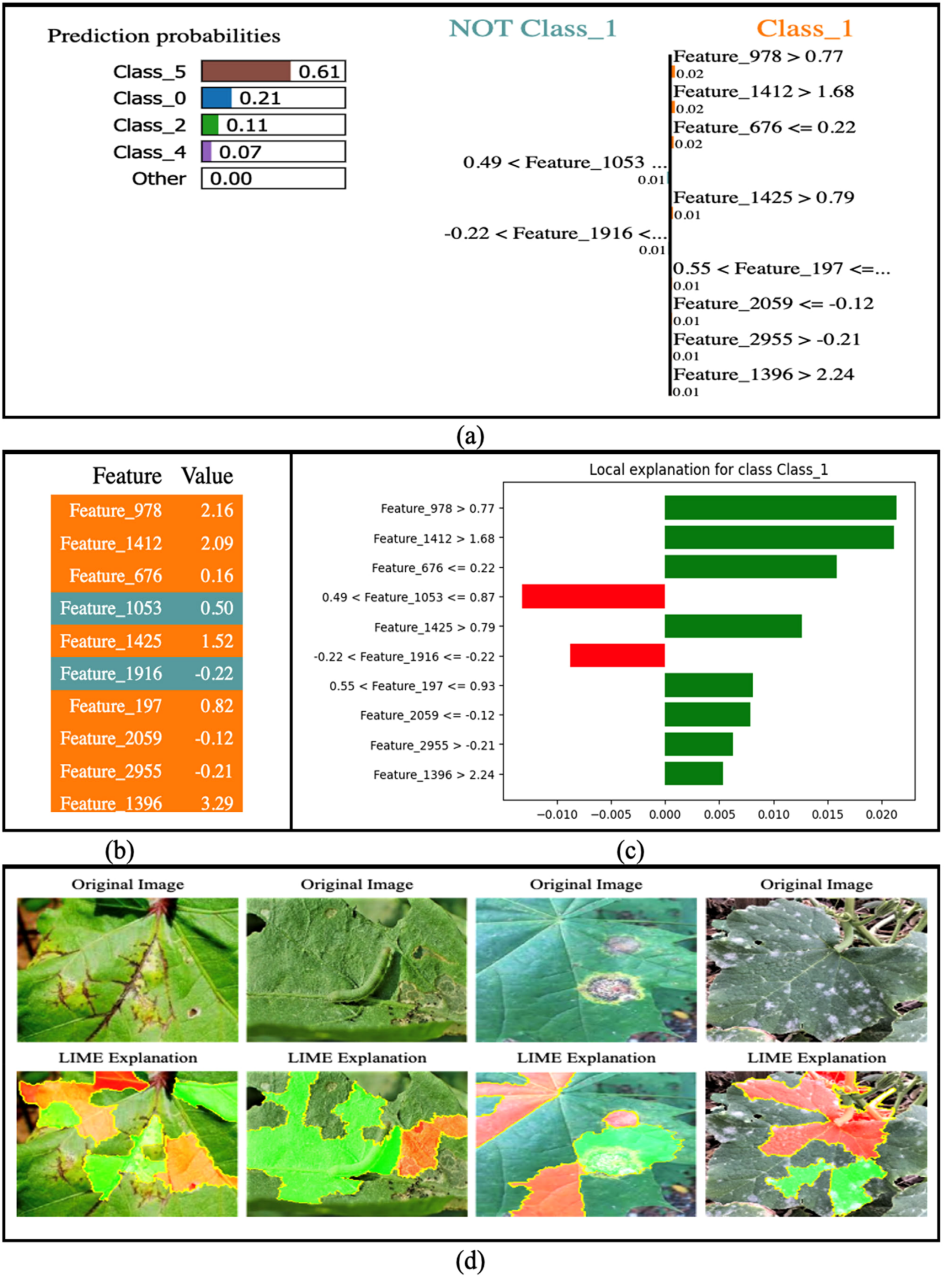
LIME visualization: (a) Prediction probabilities, (b) feature value, (c) local explanation for class 1, (d) visual explanation.

The interpretability of models is enhanced through LIME, which shows significant features that impact predictions. The system creates disruptions in the input data to examine prediction alterations for generating local explanations. The application of LIME for cotton leaf disease classification is presented in Figure 12. The model has the strongest confidence in Class_5 according to the prediction probabilities shown in Figure 12a. The important feature values behind the prediction include Feature_978 and Feature_1412, as shown in Figure 12b. The local explanation in Figure 12c provides a visual depiction of feature contributions by using bar plots to display both positive and negative values. Figure 12d offers visual explanations that demonstrate that green areas indicate positive effects on classification, but orange‐red areas lead to negative prediction results. Through the LIME approach, organizations gain confidence due to model transparency, which enables users to identify errors and make necessary adjustments. The requirement for explainable AI systems is fundamental for implementing AI‐based cotton leaf disease detection systems in operational agricultural environments.

To address the interpretability concerns and strengthen the explainability of the model, additional LIME visualizations were generated for multiple representative images from each disease class. These visualizations, now included in an extended version of Figure 12d, highlight the most influential pixel regions contributing to the classification decision. The LIME results show that the model often focuses on disease‐specific lesion patterns, discoloration zones, and vein distortions, features that align with those used by human agronomists during manual inspection. For instance, in the case of bacterial blight, the model concentrated on the water‐soaked lesion edges, while for powdery mildew, it localized to white fungal patches. These findings indicate that the model uses biologically relevant cues for decision‐making. Additionally, in a few cases, the model attended to noninformative background textures, suggesting minor overfitting, which could be mitigated through targeted data augmentation or transfer learning with attention guidance. These insights not only validate model decisions but also provide a roadmap for future training refinements and expert‐aligned learning.

**FIGURE 13:**
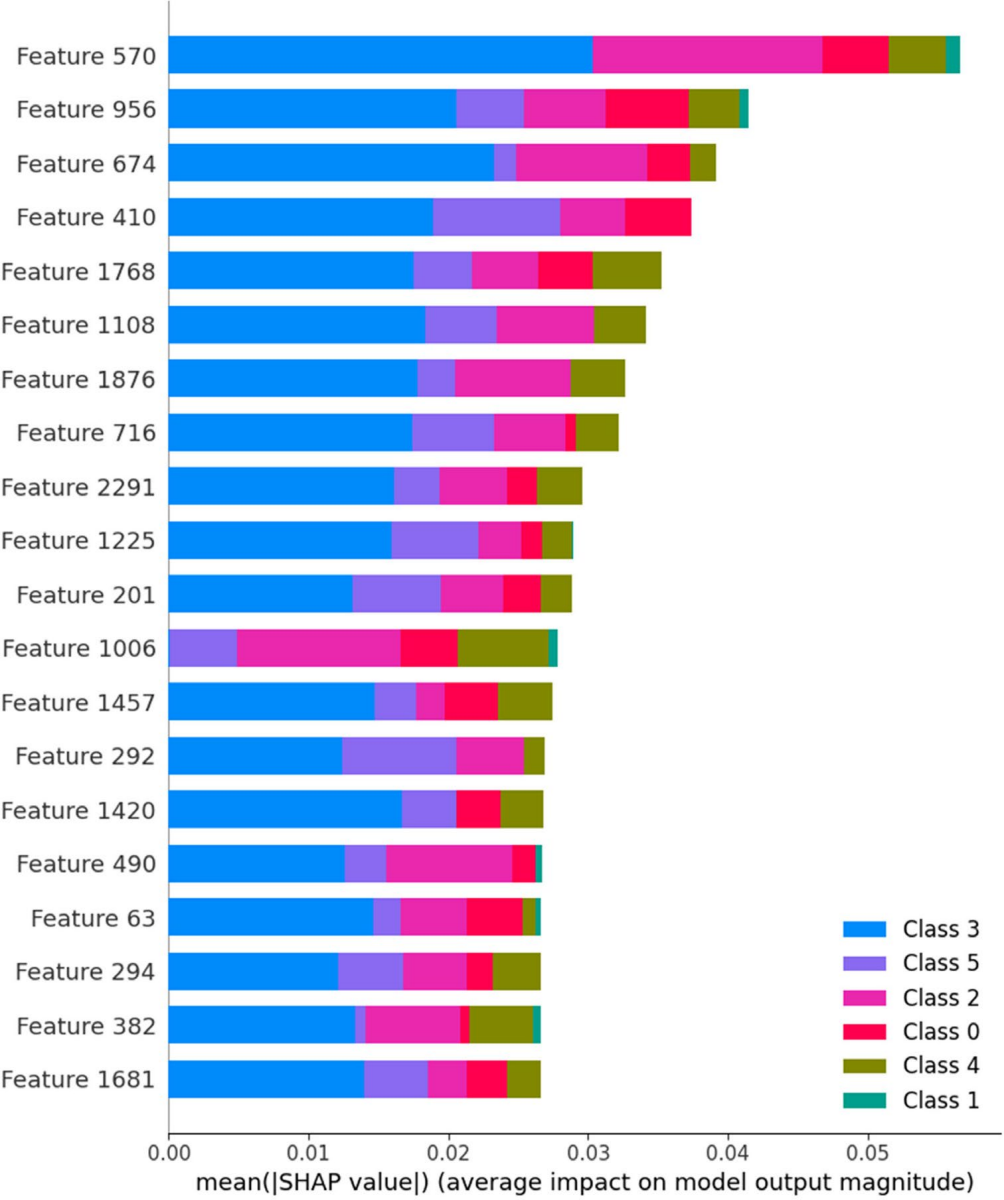
SHAP visualization.

The cutting‐edge explainability method known as SHAPs involves users whose model features make a difference in the prediction outcome. SHAP uses Shapley value computation to determine how each feature element affects the model prediction outcome. Information about the key parameters influencing different cotton leaf diseases can be found in the SHAP summary plot presented in Figure 13. Each input feature receives a measurement of importance on the basis of the assessment of mean (|SHAP value|) scores, which reflect its influence level on model predictions. The visualization reveals three main features, namely, Feature 570, Feature 956, and Feature 674, because they strongly affect the classification results, which target Class 3 red and Class 5 purple. The visual representation based on color provides quick comprehension of which characteristics play a role in which disease groups. The model's interpretability gives experts the power to understand the DL system's ability to detect diseased cotton leaves, thus enhancing their confidence in automated disease recognition solutions.

**FIGURE 14:**
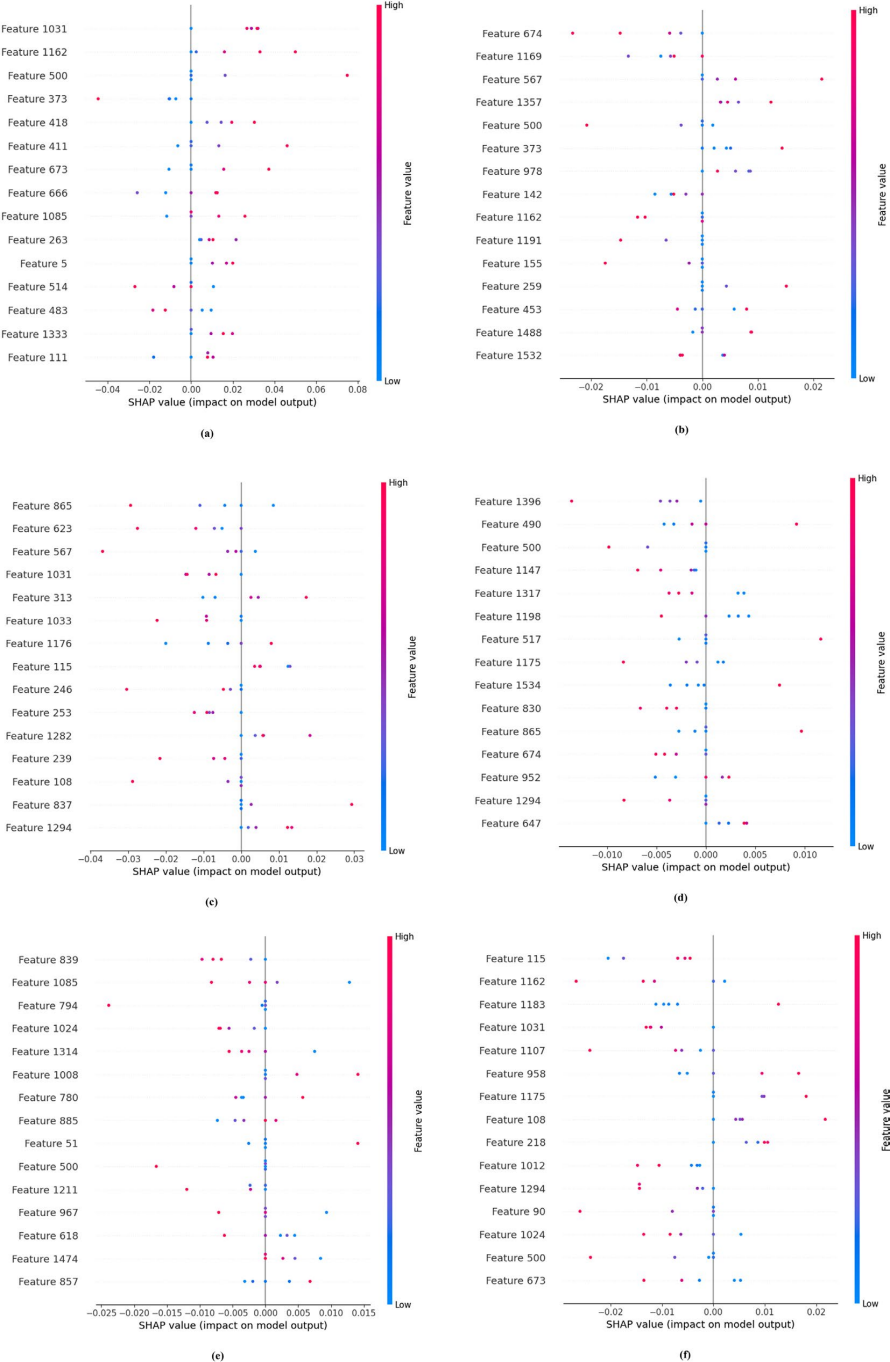
SHAP summary plots for (a) Class 0, (b) Class 1, (c) Class 2, (d) Class 3, (e) Class 4, and (f) Class 5.

Figure 14 presents SHAP summary plots for each of the six disease classes: (a) Class 0 through (f) Class 5. These plots display the top features influencing the model's prediction for each class. Each point represents an individual prediction instance, where the color indicates the feature value (red for high, blue for low), and the horizontal position indicates the SHAP value. This visualization aids in understanding class‐specific feature impact and model behavior.

## 5 State‐Of‐The‐Art Comparison

The state‐of‐the‐art models are evaluated in Table 3 to showcase their effectiveness in cotton leaf disease classification. Analysis through the table illustrates how different computer models function best depending on the dataset, which includes self‐built datasets and both the cotton disease datasets and the cotton crop leaves datasets. The CANnet model (CNN + transformer) was established as an efficient DL method because of its high accuracy performance rates of 96.3% and 98.6%, respectively. The integration of EfficientNetB7 with metaheuristic‐optimized DL produces competitive results when assessing the cotton leaf disease dataset through its optimized hyperparameter setting. The implementation of metaheuristic optimization with EfficientNet feature extraction leads to better classification robustness than both hierarchical residual networks and transformer‐based models. This research shows that evolutionary algorithm optimization of DL leads to higher detection accuracy and generalization, which deliver better results than traditional CNN‐based cotton disease detection methods do.

**TABLE 3:** State‐of‐the‐art comparison.

References	Dataset	Technique	Number of images	Number of classes	Accuracy
Ramya and Kalimuthu (2024)	Cotton leaf disease dataset	Inception V3	1710	4	97%
Uma Maheswari et al. (2025)	—	Hierarchical Residual Network‐ Red Fox Optimization (HRN‐RFO (OHRN))	2400	4	96.52%
Hyder and Talpur (2024)	Cotton disease dataset	Custom CNN VGG16 ResNet50 Proposed Model	1710	4	95.37%, 98.10%, 98.325, 90.53%
Akbar et al. (2024)	Self‐built cotton disease dataset, Public Cotton Disease Dataset	CANnet Model (CNN + Transformer)	792, 3118	3, 6	96.3%, 98.6%
Zekiwos Azath and Bruck (2021)	Cotton leaf disease dataset	CNN Model	3117	4	96.4%
Anwar et al. (2023)	Cotton disease dataset	Transfer Learning Models (Best DenseNet169 and ResNet50V2)	2654	3	96%
Nagpal and Goel (2024)	Cotton crop leaves Dataset	EfficientNetB7	2000	8	89%
Ali et al. (2025)	Plant Disease Classification Merged Dataset (subset)	Vision Transformer (ViT)	83,603 (after augmentation)	55	90%
Feng et al. (2025)	Custom‐collected outdoor dataset	LCDDN‐YOLO (Improved YOLOv8n)	6712	8	85.4%
Proposed model	Cotton leaf disease dataset	Metaheuristic‐optimized deep learning	2637	6	98%

Recent literature offers a strong foundation for advancing cotton leaf disease classification using explainable and optimized DL architectures. Zeng et al. (2025) introduced GCCNet, a gated cross‐correlation network capable of effectively leveraging multiview information for classification, a concept that can be adapted to multiperspective analysis of leaf textures and disease spread. Similarly, the MvMRL model developed by Zhang, Lin, et al. (2024) demonstrates the efficacy of multiview molecular representation learning, which supports the potential of integrating diverse data sources such as spectral and morphological leaf features in crop disease prediction tasks. From a plant biological perspective, Shan et al. (2025) emphasized the importance of m6A modifications in plant development and quality, reinforcing the agricultural significance of disease monitoring for sustaining plant health and productivity. On the technical front, Liao et al. (2024) proposed a meta‐learning‐based domain adaptation method for optical image translation, providing strategies to improve model generalization across varying cotton crop image domains. Wang et al. (2025) introduced an embedded cross‐attention framework for salient object detection, which can enhance the ability of models to localize and interpret infected regions in high‐resolution cotton leaf images. Hitimana et al. (2023) developed an intelligent DL system for identifying coffee leaf diseases using the Rwandan Arabica dataset, demonstrating the effectiveness of CNN‐based approaches in crop disease classification. Their work supports the adaptation of similar DL techniques for cotton leaf disease recognition in region‐specific agricultural datasets. Practical insights into crop disease classification using DL are provided by Bordin Yamashita and Leite (2023), who demonstrated the feasibility of edge computing for coffee disease identification, and by Ramamurthy et al. (2023), who developed a robust DL architecture tailored to Arabica coffee plants. These efforts highlight the transferable methodologies that can benefit cotton‐specific disease detection frameworks, especially in resource‐constrained rural settings. Collectively, these studies inform and justify the proposed use of metaheuristic optimization and explainability mechanisms such as attention‐based visualization and Grad‐CAM overlays to enhance the performance, reliability, and transparency of AI‐driven plant disease detection models.

## 6 Discussion

This study aimed to develop a high‐performing and interpretable DL model for multiclass cotton leaf disease classification using a hybrid architecture optimized by a metaheuristic strategy. The combination of EfficientNetB3 and InceptionResNetV2, supported by SMOTE, Focal Loss, and GA‐based hyperparameter tuning, demonstrated strong capability in accurately detecting and distinguishing between six disease classes. The proposed model achieved 98.0% accuracy, 98.1% precision, 97.9% recall, 98.0% F1 score, and an AUC‐ROC of 0.999. These results outperformed traditional CNNs and several state‐of‐the‐art models reported in the literature, validating the effectiveness of the hybrid feature extraction and GA‐based optimization approach. The model was strong, especially on underrepresented classes of diseases, where data augmentation and synthetic oversampling of data augmented with SMOTE addressed the class imbalance problems. Visualization explanation techniques like SHAP and LIME were of significant importance to increase the interpretability of the model. These methods helped to identify the most significant characteristics of the model to make predictions and to find certain region‐related reasons for each of the classes. Such interpretability is critical to actual users of the models, such as agronomists, who must be able to interpret and believe model predictions to act on such predictions in real‐world crop management systems.

Answers to Research Questions: Answers to the research questions explained in the Section on Introduction are provided below based on the results of the experiment and the clarity of the results. RQ1: The hybrid architecture (EfficientNetB3 + InceptionResNetV2) and hyperparameter tuning through GA have proved their superiority compared to standalone CNNs and other baselines introduced in the literature. It had an overall accuracy of 98%, F1‐score of 98%, and AUC of 0.9992, which indicates high classification potential in all six disease classes. The statistical tests proved that such improvements were significant (*p* < 0.05), which confirms the hypothesis that this hybrid‐GA architecture is the best one to detect cotton leaf disease. Answer to RQ2: The application of XAI tools (LIME and SHAP) provided transparent, class‐specific insights into the model's decision process. LIME highlighted localized lesion areas, while SHAP ranked features influencing predictions. These visualizations corresponded well with agronomic disease patterns, validating model behavior and making it interpretable for agricultural stakeholders. Thus, the use of XAI improved model trust and supported its real‐world deployment potential.

While the cotton leaf disease dataset used in this study (sourced from Kaggle: https://www.kaggle.com/datasets/ataher/cotton‐leaf‐disease‐dataset/data) provides a valuable and well‐organized collection of high‐quality images, it is important to acknowledge certain constraints that may affect real‐world applicability. The dataset primarily includes images captured under relatively consistent lighting conditions and may not fully represent the wide variation in illumination, background, or weather‐related artifacts encountered in diverse agricultural environments. Moreover, the images are collected from specific geographic locations, which may limit the generalizability of the model to cotton crops grown in different regions with distinct disease expressions or environmental stressors. Additionally, the dataset contains disease samples at visually identifiable stages, potentially limiting the model's ability to detect early or latent infections.

## 7 Conclusion

The authors developed an innovative, explainable DL framework for cotton leaf disease classification through the combination of metaheuristic optimization methods for better reliability and performance. The combination of the EfficientNetB3 and InceptionResNetV2 feature extractors with hyperparameter optimization from the GA enabled the model to reach superior classification accuracy. Using LIME and SHAP as XAI techniques provides interpretability through their ability to reveal significant features in model prediction results. The proposed approach offers high accuracy performance and transparency, which resolves substantial issues faced by DL‐based plant disease classification methods. Real‐world applications in smart agriculture gain significant value from the outcomes discovered in this research. The proposed automation system enables real‐time disease identification and decision‐making features for farmers and agronomists to be obtained through automated crop monitoring systems. Interpretable model outputs help transform trust in AI systems, which leads to equipped decisions for effective disease intervention control. The execution of AI models in agricultural decision‐making requires attention to ethical aspects, which focus on the reliability and accountability of the AI model. Future researchers can widen the approach by applying it to different crop disease classification cases to triangulate improved generalization capacity across several bushes and crops. One could achieve a model efficiency increase by the use of particle swarm optimization or ant colony optimization, which are metaheuristic optimization techniques. Optimization of the deployment of the model to run in real‐time on edge devices would help shape the field‐based detection of diseases, thus decreasing the reliance on the centralized computing infrastructure. The adopted innovations would help in the endorsement of digital agriculture as a promotion of sustainable and precision farming practices.

A large‐scale technical performance of the proposed model is high; however, its feasibility to be used in agricultural practices has significant challenges. The digital infrastructure is poorly accessible to everyone, especially in rural scenarios, which might hinder the utilization of real‐time AI tools in farms. Furthermore, low digital literacy among smallholder farmers might hinder the adoption process, unless suitable education and training are present. Issues of ethics also arise, in terms of how reliable and accountable AI‐driven decision‐making is to the health of crops and the livelihood of farmers being directly affected. Transparency and fairness of AI systems are very crucial, and they are facilitated by explainable AI (XAI) methods, like LIME and SHAP. The future design strategy must be farmer‐friendly, such as an easy‐to‐use mobile graphic interface, multilingual, and offline operations. It is necessary to overcome such practical and ethical issues to achieve fair accessibility and long‐term confidence with AI solutions so that they can be deployed at scale in the real world that sustains precision agriculture and rural stability.

## 8 Limitations and Future Scope

Although the proposed model exhibits high accuracy, interpretability, and efficiency, certain limitations warrant consideration and open avenues for future research. First, the model was trained and evaluated on a publicly available dataset collected under relatively uniform conditions; while effective, its generalizability to more diverse field environments (e.g., varying lighting, geography, and crop stages) may benefit from further validation using real‐world multi‐institutional datasets. Second, while LIME and SHAP were successfully used for interpretability, expanding to multimodal explainability tools and integrating expert feedback loops can further strengthen trust and transparency. Third, although the model is optimized for edge deployment, future work may explore integration with IoT systems and federated learning frameworks to support large‐scale, privacy‐preserving applications. These directions aim to enhance scalability, robustness, and real‐world adoption while preserving the model's current strengths in accuracy and interpretability.

## Author Contributions

**Gurjot Kaur:** conceptualization (equal), methodology (equal), software (equal), writing – original draft (equal), writing – review and editing (equal). **Fuad Ali Mohammed Al‐Yarimi:** methodology (equal), project administration (equal), resources (equal), writing – review and editing (equal). **Salil Bharany:** formal analysis (equal), supervision (equal), validation (equal), visualization (equal), writing – review and editing (equal). **Ateeq Ur Rehman:** conceptualization (equal), methodology (equal), project administration (equal), writing – review and editing (equal). **Seada Hussen:** data curation (equal), formal analysis (equal), validation (equal), writing – review and editing (equal).

## Ethics Statement

The authors have nothing to report.

## Consent

The authors have nothing to report.

## Conflicts of Interest

The authors declare no conflicts of interest.

## Data Availability

The dataset used in this study is publicly available at [https://github.com/gurjot000/cotton‐leaf‐disease/tree/main] and [https://www.kaggle.com/datasets/ataher/cotton‐leaf‐disease‐dataset/data].
